# Downregulation of *Gabra4* expression during alcohol withdrawal is mediated by specific microRNAs in cultured mouse cortical neurons

**DOI:** 10.1002/brb3.355

**Published:** 2015-06-02

**Authors:** Rola A Bekdash, Neil L Harrison

**Affiliations:** 1 Department of Anesthesiology, Columbia UniversityNew York, New York, 10032; 2 Department of Pharmacology, Columbia UniversityNew York, New York, 10032

**Keywords:** Alcohol withdrawal, cortex, downregulation, *Gabra4*, microRNA

## Abstract

**Background:**

Alcohol abuse and dependence are a serious public health problem. A large number of alcohol-regulated genes, (ARGs) are known to be influenced by alcohol use and withdrawal (AW), and recent evidence suggests that neuroadaptation to alcohol may be due in part to epigenetic changes in the expression of ARGs. *Gabra4,* which encodes the *α*4 subunit of GABA_A_ receptors (GABA_A_Rs), is one of a number of ARGs that show remarkable plasticity in response to alcohol, being rapidly upregulated by acute alcohol exposure. This study addressed the effects of AW on changes in the expression of *Gabra4* and related genes that encode other subunits of GABA_A_Rs, and the potential regulation of *Gabra4* by microRNAs.

**Methods:**

We studied gene and microRNAs expression, using RT-PCR and microRNA microarray in cultured cortical neurons treated with alcohol, which was then removed in order to simulate AW in vitro. We also used microRNA mimics or inhibitors, and a promoter-reporter construct carrying the 3′UTR of *Gabra4*.

**Results:**

Eleven hours after removal of alcohol, *Gabra4* was downregulated*,* with a modest increase in the expression of *Gabrg2,* but no change in the expression of *Gabra1, Gabrd,* or *Gabrb2*. microRNA profiling in neurons undergoing AW revealed upregulation in the expression of miR-155, miR-186, miR-24, and miR-375 after 8 h of AW. Transfection with molecular mimics of miR-186, miR-24, or miR-375 also downregulated *Gabra4* expression, whereas transfection with the corresponding inhibitors of these microRNAs normalized *Gabra4* expression in AW neurons to the level measured in control neurons. Promoter-reporter experiments supported the idea that miR-155, miR-186, miR-24, miR-27b, or miR-375 bind to the 3′UTR of *Gabra4* and thereby inhibit protein production.

**Conclusions:**

Our data suggest that AW decreases *Gabra4* expression, and that this may be mediated in part by the induction of specific microRNAs in cortical neurons during AW.

## Introduction

Alcohol dependence is a widespread problem that contributes greatly to health care costs and lost productivity, as well as significant mortality and morbidity. A consensus view has developed that alcohol dependence results from substantial “rewiring” of the brain in alcohol abusers, and that this process of neuroadaptation begins with changes in excitability and gene expression, and culminates in structural and functional change in the brain (Clapp et al. 2008; Koob and Volkow 2010). These neuroadaptations that are induced by chronic alcohol exposure are often manifested after the cessation of heavy alcohol use in a wide range of behavioral changes such as increased stress reactivity, extreme anxiety, sleep disruption, impaired motor function, and enhanced susceptibility to seizures (Finn and Crabbe 1997; Saitz 1998). It has become accepted that these behavioral changes observed at the organismal level are a later consequence of earlier molecular changes at the cellular and network level.

Alcohol abuse and/or withdrawal have been suggested to cause an imbalance between the activity of excitatory and inhibitory neurotransmitters in the brain. This in turn may result from alterations in the localization, trafficking, expression and/or function of receptors, including the *γ*-aminobutyric acid A receptors (GABA_A_Rs) (Devaud et al. 1997; Cagetti et al. 2003; Sanna et al. 2003; De Witte 2004; Liang et al. 2004, 2006). GABA_A_Rs are pentameric ligand-gated chloride channels (usually 2*α*, 2*β*, and *δ* or *γ*) that may be assembled from a large number of different subunit isoforms (Mody and Pearce 2004). *α*1/4/*βγ*2 receptors are located at synapses and mediate “phasic” inhibition with fast IPSPs intimately involved in the timing and synchronization of neuronal oscillations, whereas *α*1/4/6/*βδ* receptors are located extrasynaptically and mediate “tonic” inhibition in the brain, acting to regulate input resistance and membrane potential, to function as a “gain control” of output neurons (Farrant and Nusser 2005). Alteration in GABA_A_R subunit expression levels may therefore lead to changes in excitability within neuronal networks, and for this reason much attention has been devoted to mechanisms of GABA_A_R gene expression (Grabenstatter et al. 2012).

The gene that encodes the *α*4 subunit (*Gabra4*) of GABA_A_Rs shows remarkable plasticity in response to alcohol (Kang et al. 1996; Devaud et al. 1997; Mahmoudi et al. 1997; Liang et al. 2004, 2006; Suryanarayanan et al. 2011). The dramatic nature of *Gabra4* plasticity makes it an interesting candidate to be involved in the long-lasting pathological conditions of CNS hyperexcitability that are often seen in individuals that experience several episodes of alcohol withdrawal (AW). The acute upregulation of *Gabra4* is dependent on the heat shock transcription factor 1 (HSF1) (Pignataro et al. 2007), but the regulatory factors that control *Gabra4* plasticity during AW have not yet been identified.

Epigenetic mechanisms such as DNA methylation, histone modifications, and noncoding RNAs such as microRNAs (miRNAs) are now considered as major regulators of gene expression (Berger 2007; Filipowicz et al. 2008; Deaton and Bird 2011) and have been implicated as mediators of the adverse effects of alcohol on the brain (Pandey et al. 2008; Govorko et al. 2012; Ponomarev et al. 2012). Dysregulation in the expression of the epigenetic mediators known as miRNAs has been linked to stress (Babenko et al. 2012), alcohol addiction (Pietrzykowski et al. 2008; Lewohl et al. 2011; Tapocik et al. 2013), and seizures (Pichardo-Casas et al. 2012).

miRNAs are highly conserved small 18–22 nucleotide long noncoding RNAs that do not code for proteins but do regulate the expression of many protein-coding genes (Ambros 2004). These regulatory molecules can fine-tune the protein output in a very fast, efficient, and reversible manner, even in restricted neuronal compartments including dendrites and dendritic spines (Schratt 2009). Guided by the RISC complex, they usually regulate gene expression at the posttranscriptional level by recognizing the miRNA recognition element (MRE) in the3′ untranslated region (3′UTR) of their target gene. The complementarity between the seed region of miRNA (2–7 nt) and the 3′UTR of the target gene most often results in mRNA cleavage and/or in translational repression of the mRNA (Bartel 2004, 2009) thereby making these elements potentially very important players in alcohol-induced neuroplasticity. miRNAs have been shown to modulate the function of ion channels in response to alcohol exposure (Sathyan et al. 2007; Pietrzykowski et al. 2008) and to play a role in altering neuronal communication (Hsu et al. 2012), suggesting their involvement in modulating excitability within the brain. The role of miRNAs in possibly altering GABA_A_Rs subunit expression during AW has not yet been explored**.**

In this report, we studied the effects of AW on changes in expression of different subunits of GABA_A_Rs in parallel with changes in miRNA gene expression in mouse cortical neurons in culture. Our results indicate that AW downregulates *Gabra4* expression, at least in part by upregulating the expression of physiologically relevant miRNAs that can bind to MREs along the 3′UTR of *Gabra4*.

## Materials and Methods

### Primary culture of mouse cortical neurons and ethanol exposure

**Figure 1:**
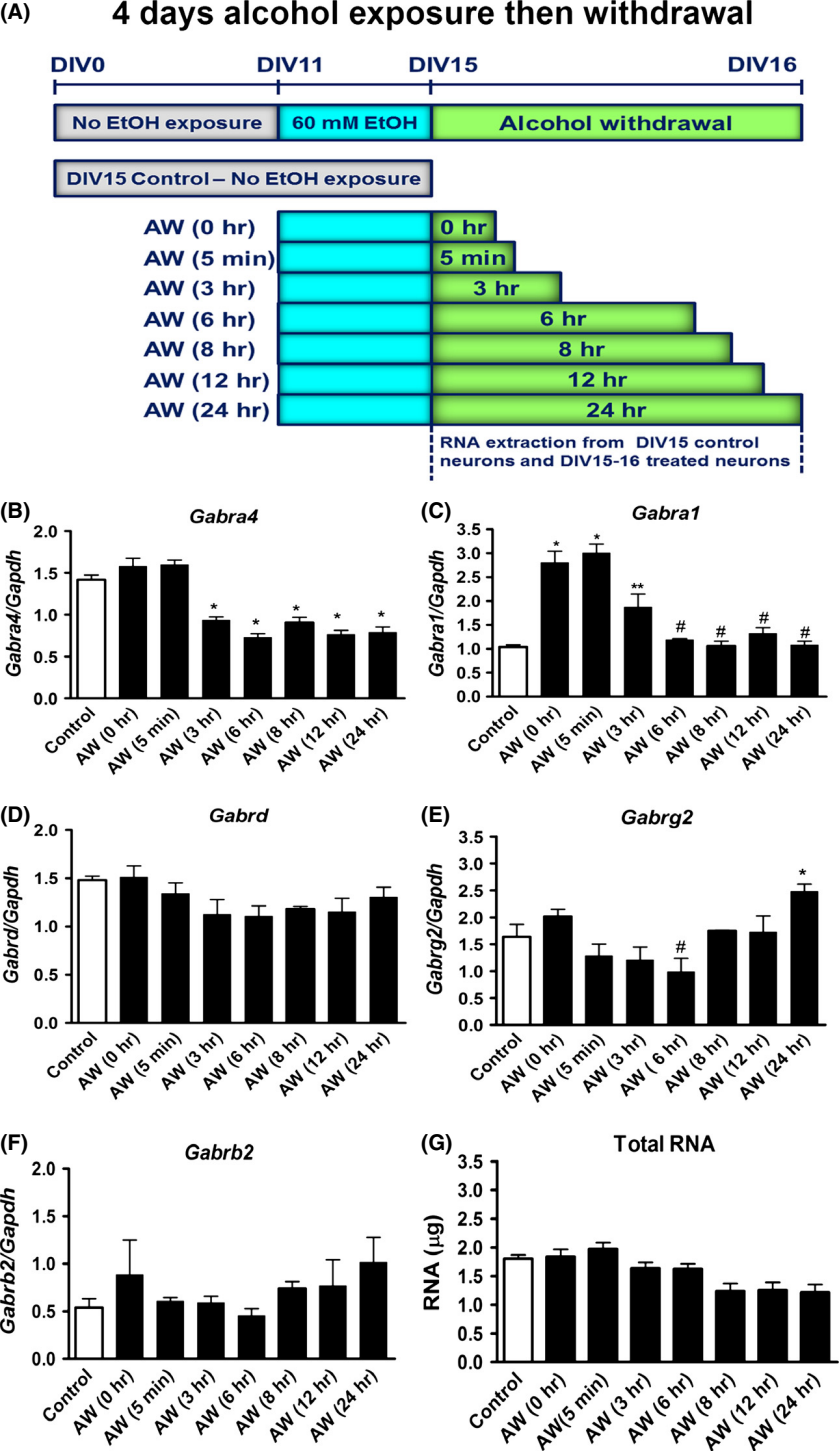
Alcohol withdrawal decreases *Gabra4* gene expression in cultured mouse cortical neurons. Effects of withdrawal from 60 mmol/L EtOH on mRNA levels of *Gabra4 (α4)*, *Gabra1 (α1)*, *Gabrd (δ), Gabrag2 (γ2),* and *Gabrb2 (β2)* in DIV15 control and DIV15–16 treated neurons. (A) Four independent cultures of primary cortical neurons were exposed at DIV11 with or without 60 mmol/L EtOH for 4 days. EtOH-treated neurons were withdrawn at DIV15 from EtOH at various time intervals (0, 5 min, 3, 6, 8, 12, and 24 h). (B) *Gabra4*; (C) *Gabra1*; (D) *Gabrd*; (E) *Gabrg2*; (F) *Gabrb2* expression in DIV15 control (C) neurons and in DIV15–16 neurons that were withdrawn from alcohol (AW) at various time intervals. Representative histograms show the mean ± SEM. *N* = 4. *Gabra4* (*P < 0.001 AW3–24 compared to C, AW0 and AW5). *Gabra1* (*P < 0.001 AW0 and AW5 compared to C; **P < 0.05 AW3 compared to C; ^#^*P* < 0.001 AW6–24 compared to AW0 and AW5). *Gabrg2* (*P < 0.05 AW24 compared to C, AW5, AW3; ^#^*P* < 0.001 AW6 compared to AW24). (G) Total amount of RNAs extracted in DIV15 control and DIV15–16 AW neurons. Data analysis was performed using one-way ANOVA and Newman–Keuls Multiple Comparison test as a post hoc test. *P* < 0.05 is considered statistically significant.

**Figure 2:**
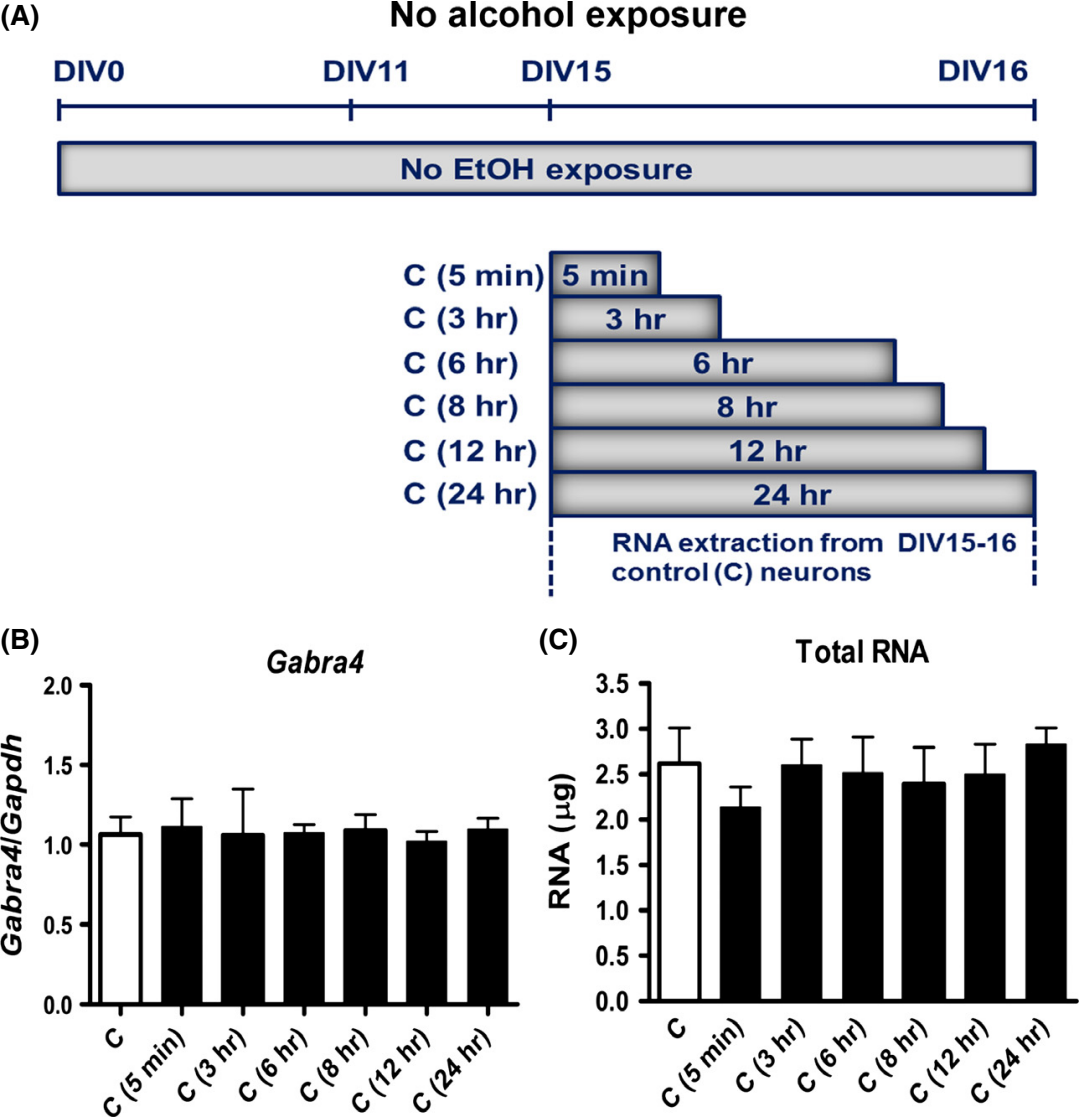
*Gabra4* gene expression was invariant in untreated DIV15–16 control neurons. (A) DIV15 untreated control neurons were incubated at various time intervals without alcohol or withdrawal. Total RNAs extraction was performed at each time point. (B) *Gabra4* expression at various time intervals (5 min, 3, 6, 8, 12, and 24 h) in untreated DIV15–16 control neurons (*N* = 3; three biological replicates; 3–5 technical replicates per each time point). (C) Total amount of RNAs extracted at various time intervals. Data analysis was performed using one-way ANOVA and Newman–Keuls Multiple Comparison test as a post hoc test.

Cortical neurons were prepared from C57BL/6J mice (Charles River) on embryonic day E18, as previously described (Ma et al. 2004) in accordance with institutional and federal guidelines. Neurons were incubated at DIV11 for 4 days in the absence or presence of ethanol (EtOHc-4D). EtOH (200 proof, Sigma, St Louis, MO) was added to culture media to reach a concentration of 60 mmol/L. Control and treated neurons (*N* = 4) were maintained in the incubator till DIV15. At DIV15, EtOH-treated cells were incubated in the absence of EtOH for additional 5 min (AW5), 3 (AW3), 6 (AW6), 8 (AW8), 12 (AW12), and 24 h (AW24). RNA extraction was performed at each time point (Fig.1A). We also performed a control experiment to determine changes in *Gabra4* expression at various time points in DIV15 control nontreated neurons (*N* = 3). RNA extraction was also performed at each time point (Fig.2A).

### Ex vivo measurements of GABA-A receptor gene expression in the cortex

Male C57BL/6J mice at postnatal day 21 (P21) were purchased from Charles River (Wilmington, MA) and maintained in environmentally controlled animal vivarium on a 12 h light/dark cycle (light on 0700 and light off 1900 h) at a constant temperature (22°C) in accordance with the requirements of the Institutional Animal Care and Use Committee of Columbia University. Mice were injected i.p. once daily from P28 for 6 days with either 3 g/kg alcohol (15% w/v) or vehicle (0.9% saline), together with 9 mg/kg of 4-methylpyrazole (4-MP; Sigma), an alcohol dehydrogenase inhibitor, to maintain high blood alcohol levels (∼60 mmol/L), similar to the concentration used in our in vitro experiments. Following the last alcohol injection, mice were withdrawn from alcohol at various time intervals and brains were collected. Tissue punches from the somatosensory cortex (SS) were pooled and collected in RNAase/DNAase-free centrifuge tubes under dry ice conditions (*N* = 10, where each represents the pooling of tissue punches from three mice). Within 24 h, RNA extraction was performed and quantitation of mRNA levels of GABA_A_Rs genes in control (C) and alcohol-withdrawn mice (AW1, AW6, AW11, and AW24) was determined.

### Quantitation of mRNA expression by quantitative real-time PCR

Total RNAs including small RNAs such as miRNAs were extracted from all groups using the miRVana miRNA isolation kit following the manufacturer’s protocol (Ambion, GrandIsland, NY) (Fig.1A). Each RNA sample was treated with RNAase-free DNAase (Qiagen, Valencia, CA) at 20–30°C for 15 min. DNAase was then heat-inactivated at 65°C for 10 min. The quantity and purity of RNAs were determined using a Nanodrop ND-1000 spectrophotometer (Thermoscientific, Waltham, MA). Ratios of A_260_/A_280_ = 1.8–2.0 and A_260_/A_230_ ≥ 1.8 were obtained for all RNA samples. The integrity and size distribution of total RNAs including smaller RNAs were assessed on 1% nondenaturing agarose gel. Two clear bands corresponding to 18S and 28S rRNA at 2:1 intensity were visible on an ethidium-bromide-stained agarose gel and no obvious degradation bands were apparent, indicating that the extracted RNA was intact (Fig. S1A, B). 500 ng of total RNAs were converted to cDNA using the iScript cDNA synthesis kit (BioRad) and the BioRad T100 Thermal cycler (Hercules, CA). The cycling conditions were as follows: 25°C/10 min, 37°C/2 h, 85°C/5 min then kept at 4°C. After the reverse transcription reaction, qPCR was performed using the Chromo 4 System following the manufacturer’s instructions (Bio-Rad). The qPCR primers (Table S1) were designed based on NCBI reference sequence database for each gene and using Primer3 software (Rozen and Skaletsky 2000). Oligos were synthesized by Integrated DNA Technologies (Coralville, IA). The amplicons were designed 80–120 bp to ensure specificity of the target gene amplification. All samples were run in duplicates and nontemplate controls were used in each run. The qPCR cycling conditions were as follows: 50°C/2 min/1 cycle; 95°C/10 min/1 cycle; 95°C/15 sec, and 60°C/1 min for 40 cycles (Life Technologies, GrandIsland, NY). A melting curve (55°C–95°C in 0.5°C increments) was performed in each run to assess the specificity of primers and to distinguish between amplicon amplification and primer-dimer formation. Relative quantitation of gene expression in control and EtOH-treated samples from four independent cultures (4 biological replicates; 3–5 technical replicates per treatment) was calculated from the ratio of the mean quantity of target gene (*Gabra4, Gabra1, Gabrd, Gabrg2, or Gabrb2*) to that of internal control (*Gapdh)*. Similarly, we determined changes in gene expression in control and alcohol-withdrawn mice. We also tested other housekeeping genes such as the *18S rRNA* and *β-actin,* but found that *Gapdh* is the most appropriate housekeeping gene to use for this study as its expression did not change significantly between control and treated groups.

### miRNA expression profiling and stem loop real-time PCR

We used TaqMan Low Density Array Card A V.2 (TLDA; Life Technologies) to profile changes in miRNAs expression in DIV15 control, DIV15 EtOHc-4D, and DIV15 AW (8 h) neurons. RNA samples (total of 12, four per group) were each hybridized to a card that contains 374 known mouse miRNA probes from the Sanger miRBase V15 (http://microrna.sanger.ac.uk). Briefly, 500 ng of enriched miRNAs were reverse transcribed using the Megaplex RT primers Rodent Pool A and the TaqMan miRNA RT kit following the manufacturer’s instructions (Life Technologies). qPCR was performed using the ABI Prism 7900 HT system. TLDA thermal cycling conditions were as follows: 50°C/2 min, 94.5°C/10 min, 40 cycles of 97°C/30 sec, 59.7°C/1 min. We validated the results of microarray data for selected miRNAs with stem-loop RT-PCR analysis using TaqMan miRNA assays (Chen et al. 2005). The endogenous control U6 snRNA was used for normalization. The expression of U6 did not change between control and treated neurons. Comparison in expression between groups was made using the 2^-ΔΔCT^ method.

### Transfection of miRNA mimics or inhibitors into cultured mouse cortical neurons

Cortical neurons were maintained in culture as previously described (Ma et al. 2004). We quantitatively assessed transfection efficiency of mimics or inhibitors using miRNA mimic, miR-1 Positive Control (PC) and Negative Control NC # 1 and miRNA inhibitor let-7c PC and NC #1 (Ambion), and determined their effects on changes in *Ptk9 or Hmga2* expression (Fig. S2). We used 30 nmol/L of mimic (Fig. S2A, B) but 100 nmol/L of inhibitor (Fig. S2C, D) as lower concentrations of the inhibitor (10–50 nmol/L) did not elicit an effect on *Gabra4* expression. The sequence of upregulated miRNAs (miR-155, miR-186, miR-24, and miR-375) from TLDA card was verified as the 5p or 3p strand using www.mirbase.org (Griffiths-Jones et al. 2008). Accordingly, mimics or inhibitors to the specified miRNA were designed (Life Technologies). Cortical neurons were seeded at 10^6^ cells/mL in a 12-well plate then transfected at DIV8 for 2–3 h with mimic or inhibitor of miR-155, miR-186, miR-24, or miR-375 following the manufacturer’s instructions. For normalization of transfection efficiency, control and EtOH-treated neurons were transfected with 30 nmol/L of BLOCK-iT Alexa Fluor Red Fluorescent Oligo (Life Technologies) which has a sequence that is not homologous to any known gene. Transfection was then performed using Lipofectamine RNAIMAX reagent following the manufacturer’s instructions (Invitrogen, GrandIsland, NY). Transfected neurons were incubated at 37°C for 2–3 h. After 3 h transfection, transfection medium was removed and replaced with conditioned medium. Neurons were then maintained at 37°C prior to EtOH treatment. At DIV11, transfected neurons were incubated in the absence or presence of 60 mmol/L EtOH for 3 days not for 4 days as the activity of mimics or inhibitors lasts for 11 days after transfection. At DIV14, transfected neurons were incubated for additional 8 h in the absence of EtOH. Quantitation of *Gabra4* expression in transfected neurons was determined by qPCR as previously described.

### Cloning the 3′UTR of the *Mus musculus* Gabra4 into a pMIR-REPORT vector

The sequence of the 3′UTR of *Mus musculus Gabra4* (1965 bp) was obtained from Ensembl database (www.ensembl.org) and cloned into pMK-RQ vector (4253 bp) by GeneArt (Life Technologies). The construct was then verified by sequencing. Restriction sites for *Sac* I and *Hind* III were included at the 5′- and 3′-end of Gabra4 3′UTR sequence (Fig. S3A). The 3′UTR of *Mus musculus* Gabra4 plasmid (pMK-RQ, 4253 bp) was then digested by *Sac* I and *Hind* III (Fig. S3B) (Invitrogen) and subcloned into a pMIR-REPORT luciferase vector (pMIR-Luc) (Life Technologies, AM5795). We also used *β*-Galactosidase control vector (pMIR-Gal). Gabra4 3′UTR expression vector (pMIR-Luc-3′UTR) was then transformed into chemically competent TOP10 *E. coli* cells following the manufacturer’s instructions (Life Technologies). The efficiency of transformation was assessed using the pUC19 control plasmid. Plasmid DNA was extracted from white colonies that were grown overnight in Luria broth supplemented with 10 mg/mL ampicillin using the PureLink HiPure plasmid DNA purification kit (Life Technologies). Successful transformation of *E. coli* with pUC19, pMIR-Luc-3′UTR or with pMIR-Gal plasmids was assessed by PCR reaction. The following primers were designed using Primer3 software to amplify a region (533 bp) of the 3′UTR of Gabra4 (F: 5′-CCTCACAGCCTGGAACCTAC-3′; R: 5′-CACAGAATCTTGCGAGGACA-3′). A typical PCR reaction contains 25 *μ*L of Premix Ex Taq Hot Start Polymerase (Takara Bio Inc, MountainView, CA), DNA template, 0.1 *μ*mol/L forward, 0.1 *μ*mol/L reverse primers and nuclease-free water. PCR cycling conditions were as follows: 94°C/10 min/1 cycle; 95°C/30 sec, 58°C/30 sec, 72°C/30 sec for 40 cycles; 72°C/10 min (Takara Bio Inc). The size of the amplified PCR product was assessed on nondenaturing 1% agarose gel stained with ethidium-bromide (Invitrogen) and using TrackiT 1 Kb Plus DNA ladder (Life Technologies) (Fig. S3D). pMIR-Luc-3′UTR was digested with HindIII/SacI and the digests were run on 1% agarose gel (Fig. S3C). The subcloning of Gabra4 3′UTR into a pMIR-REPORT vector was further confirmed by sequencing the isolated plasmid using primers that amplify a 533 bp of the Gabra4 3′UTR and Genewiz universal primers that amplify a region of the SV40 promoter (20 mer 5′-d(TATTTATGCAGAGGCCGAGG)-3′) and the CMV promoter (21 mer 5′-d(CGCAAATGGGCGGTAGGCGTG)-3′) of pMIR-Luc vector (6470 bp).

### Dual luciferase reporter assay

Dual luciferase reporter assay was performed following the manufacturer’s instructions (Life Technologies). To assess the effect of selected miRNAs on changes in Gabra4 protein levels, we performed ELISA following the manufacturers’ instructions (Cedarlane/Lifeome). The detailed methods are described in “Data S1″.

### Immunostaining for *α*4 peptide levels in cultured mouse cortical neurons

Dissociated cortical neurons, derived from three independent neuronal cultures, were grown on N^o^1 German 15 mm in diameter glass coverslips (Fisher Scientific, Waltham, MA) precoated overnight with 0.05 mg/mL of Poly-D-lysine hydrobromide (Sigma) then washed with sterile distilled H_2_O and incubated at least for 2 h at 37°C before plating as previously described with minor modifications (Kaech and Banker 2006). Cultured cortical neurons grown on coverslips in 12-well plates (Corning) were incubated at DIV11 for 4 days in the absence or presence of 60 mM EtOH. At DIV15, ethanol-treated neurons were incubated for additional 8 h in the absence of EtOH. DIV15 control and DIV15 AW (8 h) neurons were stained for the *α*4 subunit as previously described with minor modifications (Jia et al. 2005). We used four coverslips per treatment in each independent experiment. Briefly, neurons were fixed with 4% PFA (15 min). After fixation, neurons were permeabilized with 0.3% TritonX100 in PBS (1X) (5 min), washed twice with PBS (1X) (5 min) then blocked for 1 h at room temperature with 10% donkey horse serum (VectorLabs, Burlingame, CA). After blocking with horse serum, neurons were stained with rabbit polyclonal antibody (Ab) to *α*4 subunit of GABA_A_Rs at 4°C overnight (1:500) (Novus Cat# NB300-194, RRID: AB_2109118). The secondary Ab Alexa Fluor 594 donkey anti-rabbit IgG (1:500/45 min) (Life Technologies Cat# A21207, RRID: AB_10049744) was used. After incubation with secondary Ab, neurons were then washed twice with PBS (1X) and mounted in Vectashield mounting media (VectorLabs). As a negative control, omission of the primary Ab from the staining procedure did not show significant immunoreactivity signal.

### Confocal microscopy and image analysis

Fluorescent images of cortical neurons were captured using a Nikon A1 scanning confocal microscope on an Eclipse Ti microscope stand (Nikon Instruments, Melville, NY). All images were taken using a 40X/1.3 Plan-Fluor objective lens. Standard lasers and filters were used to image DAPI and TRITC. The confocal pinhole was set at 1 Airy unit to produce an optical section ∼0.5 *μ*m thick. All scanning settings were held constant throughout the experiment. To maintain consistency, for each field analyzed, a single optical section was collected from the middle focal plane of neuronal cells using Z stack. The middle was defined as the plane equidistant from the top and bottom surfaces of these cells. We randomly chose 8–10 different regions per coverslip for quantification.

Images were analyzed using NIS Elements 4.0 (Nikon). To quantify the total integrated intensity of the *α*4 subunit (TRITC channel) in control and AW8 neurons, we manually defined a threshold range for this channel. The thresholded pixels were subjected to one iteration of the Elements software’s “Clean” function to remove stray pixels and one iteration of the “Smooth” function to reduce noise at the edges of detected structures. The threshold of this channel, and the processing steps, were kept constant throughout the experiment. The processed mask was then used as regions of interest (ROI) to measure the total integrated intensity of *α*4 subunits.

### Statistical analysis

Statistical analysis of data was performed using Graph Pad Prism version 4.0 (LA Jolla, CA). For comparison of mRNA expression between groups, we used one-way ANOVA and Newman–Keuls multiple comparison test as a *post hoc* test. The analysis of microarray data and luciferase assay were performed using Student’s *t*-test. miRNA expression analysis was performed using two-way ANOVA and Bonferroni as a *post hoc* test or Student’s *t*-test. Analysis of immunostaining data was performed using the paired *t*-test. All results are presented as standard error of the mean (±SEM) or standard deviation (SD). *P* < 0.05 is considered statistically significant.

## Results

### AW downregulates *Gabra4* expression in cultured mouse cortical neurons

We determined changes in mRNA expression of GABA_A_R subunits (*α4, Gabra4; α1, Gabra1; δ, Gabrd, γ2, Gabrg2; β2, Gabrb2)* in DIV15 control neurons and in neurons that were withdrawn from alcohol (AW) at various time intervals in cultured mouse cortical neurons. EtOH concentration used in this study (60 mmol/L) is at the high end of the range associated with intoxication, but is tolerated by chronic alcoholics (Urso et al. 1981). We found that DIV15-16 neurons had a significant decrease in *Gabra4* expression between 3 and 24 h after the onset of AW (AW3-AW24) compared to DIV15 control neurons (Fig.1B). *Gabra1* expression increased significantly at 0 and 5 min after the onset of AW (AW0-AW5), and at AW3 compared to controls then reverted back to normal at (AW6-24) (Fig.1C). *Gabrd* (Fig.1D) and *Gabrb2* (Fig.1F) expression did not change but *Gabrg2* expression was upregulated at AW24 (Fig.1E). The amount of total RNA extracted (∼1.5 *μ*g/mL) did not differ significantly between groups (Fig.1G).

To rule out the possibility that the changes in *Gabra4* expression we observed were due to circadian or other cyclical factors rather than to AW, we also monitored changes in *Gabra4* expression at various time intervals in DIV15 control nontreated neurons that were not exposed to alcohol (Fig.2A). We found that *Gabra4* expression and total RNA extracted did not change significantly over time (Fig.2B, C).

### AW downregulates *Gabra4* expression in the mouse somatosensory cortex

**Figure 3:**
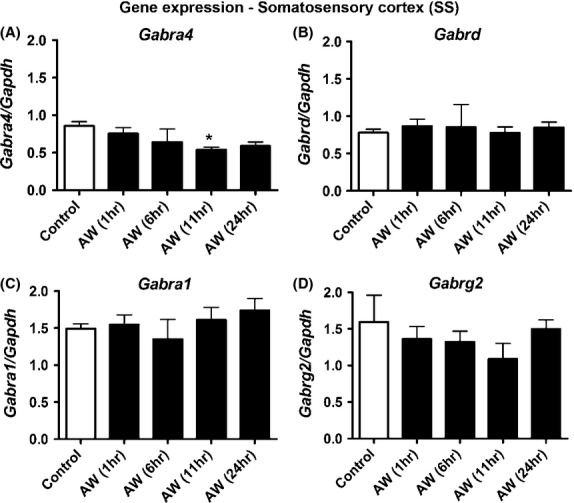
AW decreases *Gabra4* expression in the mouse somatosensory cortex. Effects of AW on changes in (A) *Gabra4*; (B) *Gabrd*; (C) *Gabra1*; (D) *Gabrg2* expression in the somatosensory cortex of control (C) and alcohol-withdrawn mice (AW1, 6, 11, and 24 h) (*N* = 10, each *N* represents the pooling of tissues from three mice). Representative histograms show the mean (±SEM). *Gabra4* (*P < 0.05 AW11 compared to C). Data analysis was performed using one-way ANOVA and Newman–Keuls Multiple Comparison test as a post hoc test. *P* < 0.05 is considered statistically significant.

We also determined changes in mRNA expression of GABA_A_R subunits (*α4, Gabra4; α1, Gabra1; δ, Gabrd, γ2, Gabrg2)* in the somatosensory cortex of control and alcohol-withdrawn mice. We identified 11 h after the onset of AW as the peak of behavioral withdrawal at which point mice showed mild seizure-like symptoms (Hale et al. 1992; Farook et al. 2008). We found that *Gabra4* expression (Fig.3A) was reduced at AW 11 h with no significant changes in the mRNA levels of *Gabrd, Gabra1,* or *Gabrg2* (Fig.3B–D).

### Chronic exposure to alcohol and subsequent withdrawal alter miRNA expression profile in cultured mouse cortical neurons

**Figure 4:**
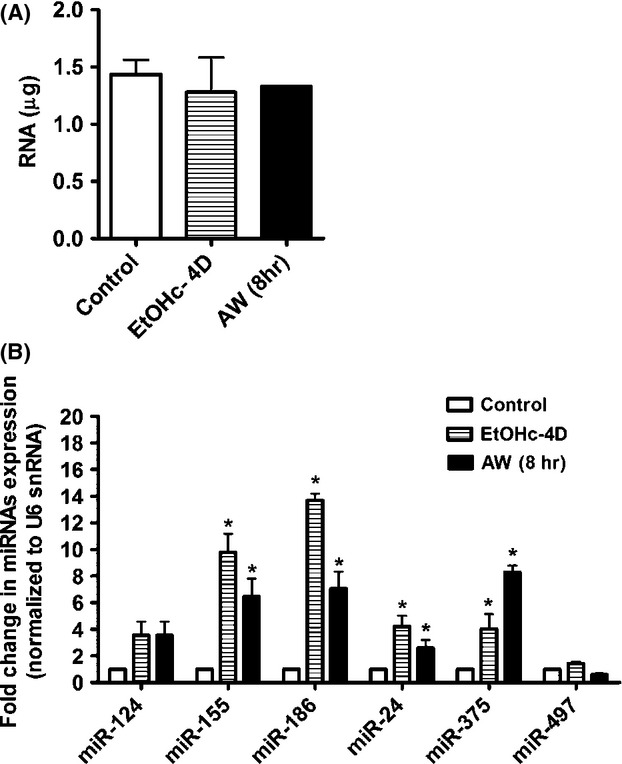
Chronic exposure to alcohol and withdrawals upregulate the expression of selected miRNAs in cultured mouse cortical neurons. (A) Total amount of RNA extracted from DIV15 control, DIV15 EtOHc-4D, and DIV15 AW (8 h) neurons. (B) qRT-PCR analysis confirmed the upregulation in expression of miR-155, miR-186, miR-24, and miR-375 in EtOHc-4D and/or AW (8 h) neurons. miR-124 is a positive control gene and miR-497 is a negative control gene. Data analysis of miRNA gene expression was performed using two-way ANOVA and Bonferroni as a post hoc test. *N* = 4; *P < 0.05 is considered statistically significant.

miRNA microarray analysis was performed using TLDA Card to profile changes in miRNAs gene expression in control neurons (C, *N* = 4), in neurons that were exposed to 60 mmol/L EtOH for 4 days (EtOHc-4D, *N* = 4) and in neurons that were withdrawn from chronic exposure to 60 mmol/L EtOH for 8 h (AW8, *N* = 4). RNA extraction was performed at DIV15 in control and treated neurons. The results of four independent experiments showed that 18 miRNAs in EtOHc-4D neurons (Table S2) and 22 miRNAs in AW8 neurons (Table S3) were significantly upregulated. The amount of total RNA extracted from all groups is represented in Figure4A. The array data revealed that ten miRNAs were significantly upregulated in both EtOHc-4D and in AW8 neurons in comparison to control neurons. A number of miRNAs were undetectable in all groups, which indicates very low or no expression in the mouse cortex. Two miRNAs, miR-10b, and miR-873, were significantly downregulated in EtOHc-4D and AW8 neurons (data not shown).

### Chronic exposure to alcohol and subsequent withdrawal upregulate the expression of specific miRNAs in cultured mouse cortical neurons

**Figure 5:**
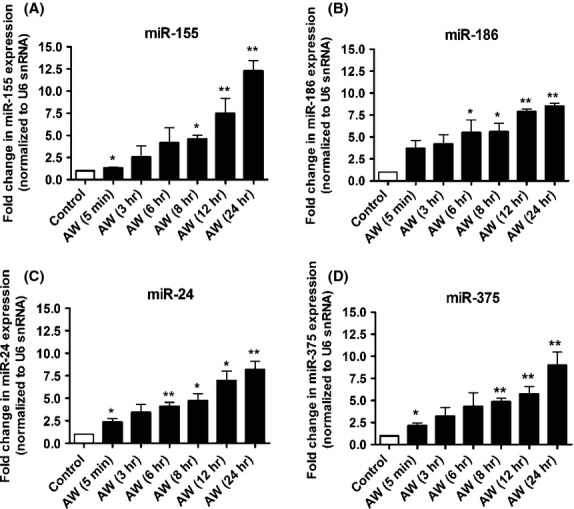
Time course of upregulation of miR-155, miR-186, miR-24, and miR-375 gene expression during AW in cultured mouse cortical neurons. Fold change in miRNA gene expression in DIV15 control neurons (C) and in DIV15–16 neurons withdrawn from EtOH at various time intervals (5 min, 3, 6, 8, 12, and 24 h). *N* = 3 (three independent neuronal cultures; 3–5 technical replicates per time point). (A) miR-155 (*P < 0.01 AW5 and AW8 compared to C, **P < 0.05 AW12 and AW24 compared to C), (B) miR-186 (*P < 0.05 AW6 and AW8 compared to C, **P < 0.01 AW12 and AW24 compared to C), (C) miR-24 (*P < 0.05 AW5, AW8 and AW12 compared to C; **P < 0.01 AW6 and AW24 compared to C), (D) miR-375 (*P < 0.05 AW5 compared to C, **P < 0.01 AW8, AW12 and AW24 compared to C). Data analysis was performed using the paired *t*-test.

We confirmed changes in expression of selected miRNAs (miR-155, miR-186, miR-24, miR-375) by qPCR. The results of qPCR were generally consistent with microarray data (Fig.4B). The expression of miR-124, a widely expressed miRNA in the mouse brain and miR-497 that is mainly enriched in the cerebellum (Sempere et al. 2004; Bak et al. 2008), were not altered significantly in this study (Fig.4B). Because our miRNA array data showed that the upregulation of some miRNAs persisted in AW8 neurons after the removal of alcohol, we reasoned that the downregulation in *Gabra4* during AW might be mediated by specific miRNAs. We determined changes in miR-155, miR-186, miR-24, and miR-375 expression at various time intervals after onset of AW, and found that these miRNAs were significantly upregulated at AW5 min, AW6, AW8, AW12, and/or AW24 h (Fig.5A–D).

### Transfection of mimics of miR-186, miR-24, and/or miR-375 downregulates *Gabra4* expression

**Figure 6:**
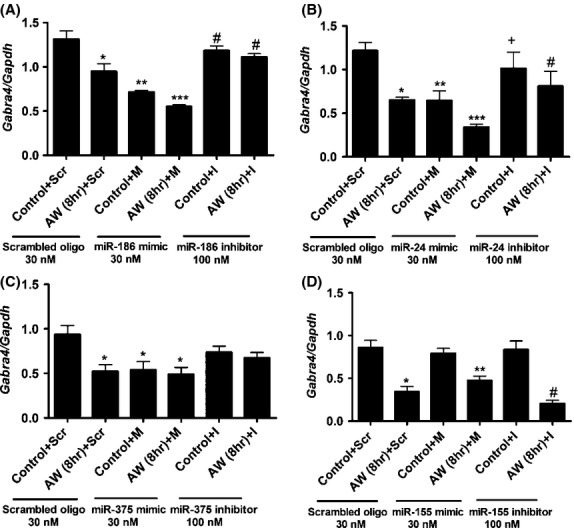
Transfection of mimics to miR-186, miR-24, or miR-375 into cortical neurons downregulates *Gabra4* expression during AW. Effects of miRNA mimic (M) or inhibitor (I) on changes in *Gabra4* expression during AW. Control neurons (C) or AW8 neurons were transfected with scrambled oligos (30 nmol/L), mimic (30 nmol/L) or inhibitor (100 nM) of (A) miR-186, (B) miR-24, (C) miR-375, (D) miR-155. *N* = 3 (miR-186; *P < 0.01 AW8 + Scr compared to C + Scr, **P < 0.001 C + M compared to C + Scr, **P < 0.01 C + M compared to AW8 + Scr, C + I, AW8 + I, ***P < 0.001 AW8 + M compared to C + Scr, ***P < 0.01 AW8 + M compared to AW8 + Scr, ^#^*P* < 0.001 C + I and AW8 + I compared to AW8 + M) (miR-24; *P < 0.01 AW8 + Scr compared to C + Scr, **P < 0.01 C + M compared to C + Scr,***P < 0.001 AW8 + M compared to C + Scr, ^+^*P* < 0.01 C + I compared to AW8 + M, ^#^*P* < 0.05 AW8 + I compared to C + Scr and AW8 + M) (miR-375; *P < 0.05 AW8 + Scr, C + M and AW8 + M compared to C + Scr) (miR-155; *P < 0.001 AW8 + Scr compared to C + Scr, C + M, C + I, **P < 0.01 AW8 + M compared to C + M, C + I, ^#^P < 0.001 AW8 + I compared to C + Scr, C + M, C + I, ^#^*P* < 0.05 AW8 + I compared to AW8 + M). Data analysis was performed using one-way ANOVA and Newman–Keuls Multiple Comparison test as a post hoc test.

We assessed the effects of mimics or inhibitors of miR-155, miR-186, miR-24, or miR-375 on changes in *Gabra4* expression in control and AW8 neurons. We found that miR-186 mimic (M) significantly downregulated *Gabra4* expression in AW8 + M neurons compared to control neurons (control + Scr) or AW neurons that were transfected with a scrambled oligo (AW8 + Scr). Conversely, a miR-186 inhibitor normalized *Gabra4* expression to a level comparable to that seen in control neurons (Fig.6A). miR-24 mimic significantly reduced *Gabra4* expression in AW8 + M neurons and its corresponding inhibitor normalized its expression (Fig.6B). miR-375 mimic reduced *Gabra4* expression, but this reduction was similar to that observed in AW8 + Scr neurons (Fig.6C). Changes in *Gabra4* expression in AW8 neurons that were transfected with miR-155 mimics were comparable to the changes in *Gabra4* expression in AW8 + Scr neurons. miR-155 inhibitor did not normalize *Gabra4* expression compared to control neurons (Fig.6D).

### miRNAs mimics decrease luciferase reporter activity in cultured mouse cortical neurons

**Figure 7:**
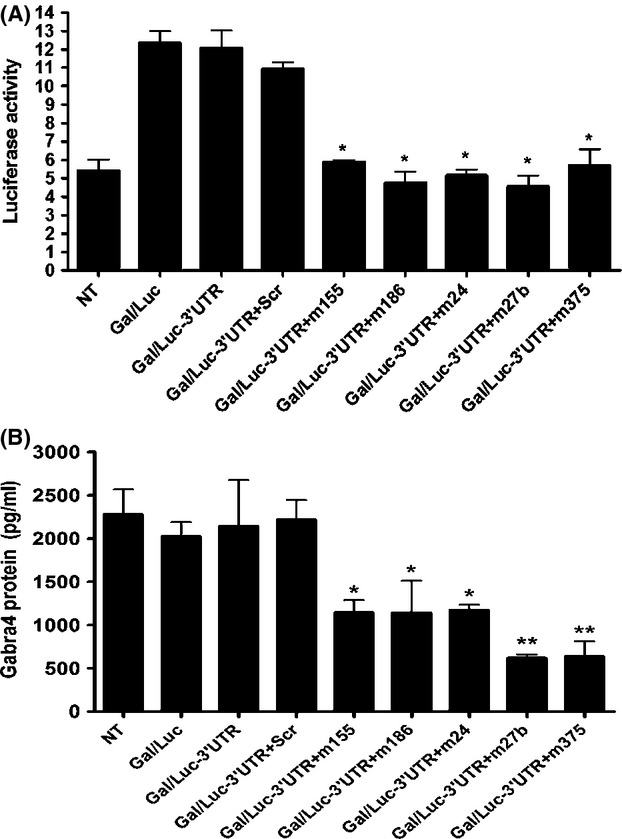
Transfection of miR-155, miR-186, miR-24, miR-27b, or miR-375 mimics decreases luciferase activity and Gabra4 protein levels in cultured mouse cortical neurons. (A) Primary cortical neurons were cotransfected at DIV8 with Gal/Luc, Gal/Luc-3′UTR plasmids in the absence or presence of scrambled oligo (Scr; 30 nmol/L) or miRNA mimic (30 nmol/L) of miR-155 (m155), miR-186 (m186), miR-24 (m24), miR-27b (m27b), or miR-375 (m375). NT = nontransfected neurons. *N* = 4 (*P < 0.05 Gal/Luc-3′UTR + m155/m186/m24/m27b/m375 compared to Gal/Luc, Gal/Luc-3′UTR, and Gal/Luc-3′UTR + Scr). (B) Gabra4 protein level was significantly decreased in neurons transfected with miRNA mimic (m155, m186, m24, m27b, or m375) compared to NT, Gal/Luc, Gal/Luc-3′UTR, or Gal/Luc-3′UTR + Scr (*N* = 3) (*P < 0.05 or **P < 0.01). Data analysis was performed using one-way ANOVA and Newman–Keuls Multiple Comparison test as a post hoc test.

To determine whether any of the upregulated miRNAs could bind to the 3′UTR of Gabra4, we performed a luciferase reporter assay. Cortical neurons were cotransfected with pMIR-Gal + pMIR-Luc (Gal/Luc), pMIR-Gal + pMIR-Luc-3′UTR in the absence (Gal/Luc-3′UTR) or presence of scrambled oligo (Gal/Luc-3′UTR + Scr) or mimic of miR-155 (m155), miR-186 (m186), miR-24 (m24), miR-27b (m27b), or miR-375 (m375). Luciferase activity was significantly reduced in neurons transfected with mimics to a level comparable to that of nontransfected neurons (NT). Cortical neurons transfected with Gal/Luc-3′UTR + Scr showed a change in luciferase activity that is similar to that of neurons that were transfected with Gal/Luc or Gal/Luc-3′UTR (Fig.7A).

Using the same lysates obtained for the luciferase assay, we performed ELISA and found that Gabra4 protein level was significantly attenuated in neurons that were transfected with mimics compared to NT neurons or to neurons that were transfected with Gal/Luc, Gal/Luc-3′UTR, or Gal/Luc-3′UTR + Scr (Fig.7B).

### AW decreases total protein levels of *α*4 in cultured mouse cortical neurons

**Figure 8:**
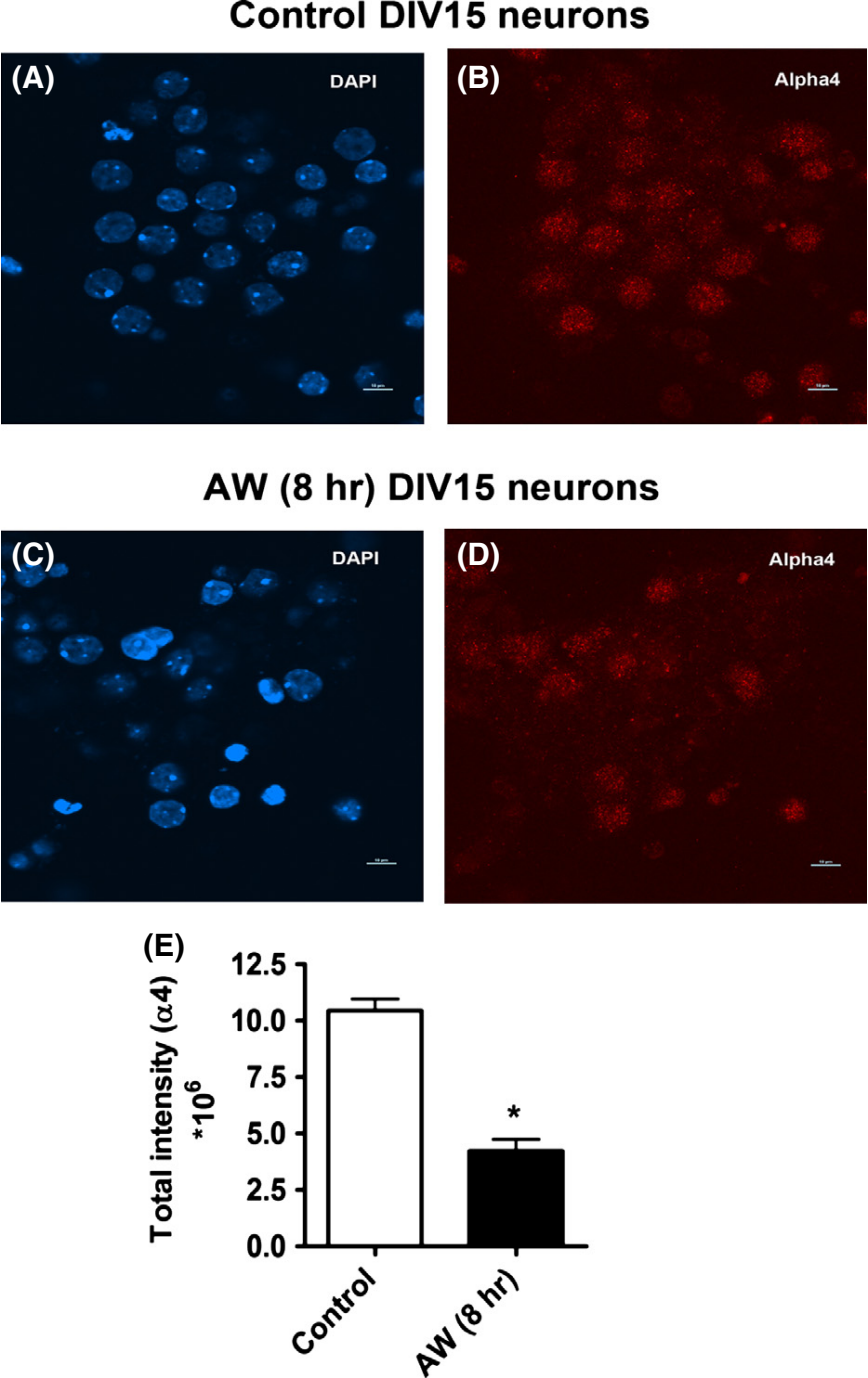
Alcohol withdrawal decreases *α*4 immunoreactivity in cultured mouse cortical neurons. Immunostaining of the *α*4 subunit of GABA_A_Rs in DIV15 control neurons (A and B) and DIV15 AW (8 h) neurons (C and D). (E) The total integrated intensity of *α*4 in control and AW (8 h) neurons. *N* = 3 (*P < 0.05 AW (8 h) compared to control). Data analysis was performed using the paired *t*-test.

We also determined the effects of AW on changes in total protein levels of *α*4 using immunostaining. Representative pictures of *α*4 immunoreactivity in DIV15 control and DIV15 AW8 neurons are presented in Figure8A–D. Total intensity of *α*4 subunit peptide levels was significantly reduced in AW8 neurons compared to control neurons (Fig.8E).

### Analysis of predicted miRNA targets and biological pathways

**Figure 9:**
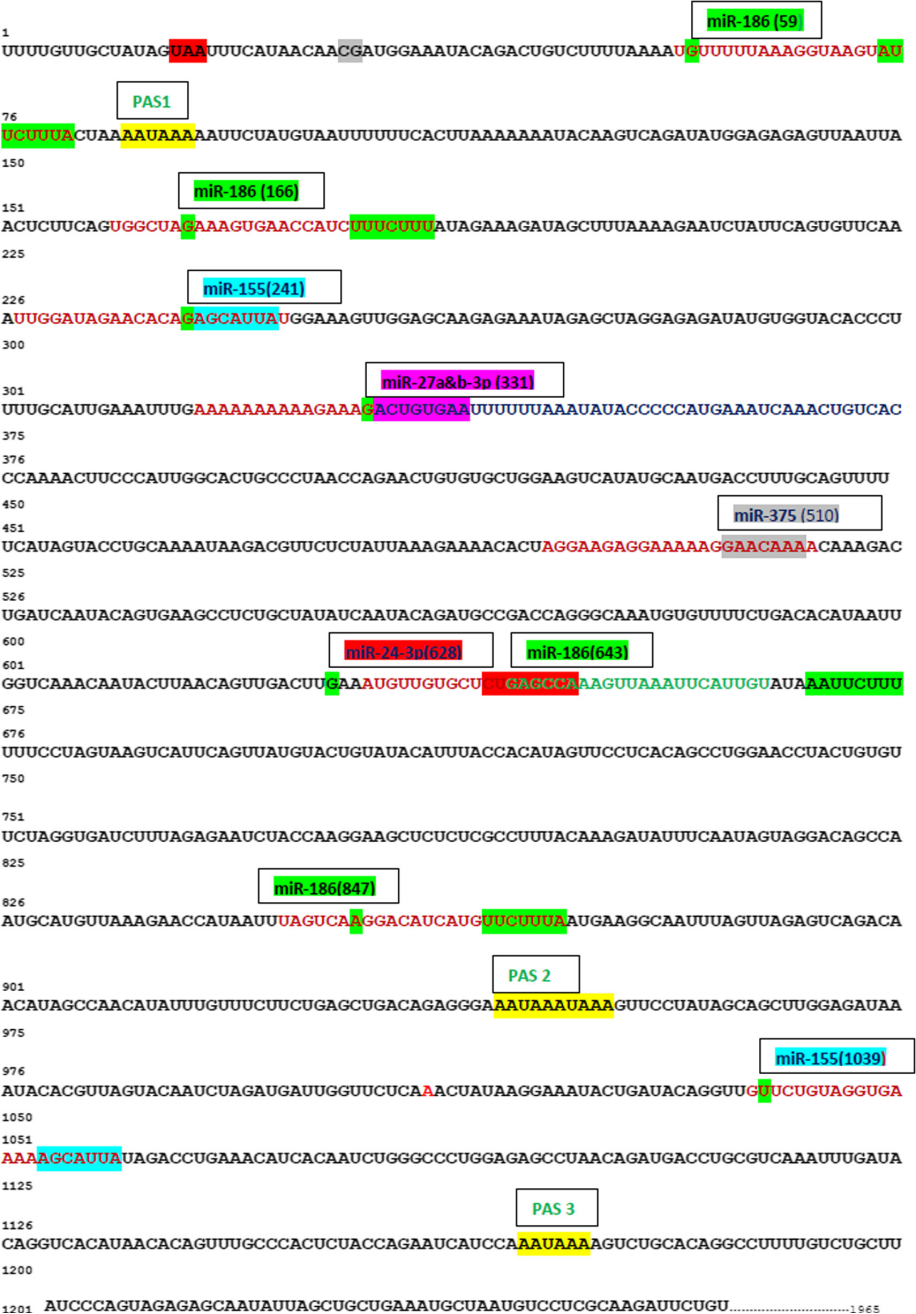
miRNAs recognition elements (MREs) along the 3′UTR of the *Mus musculus Gabra4*. miR-155, miR-186, miR-24, miR-27b, and miR-375 have predicted binding sites along the 3′UTR of *Gabra4* (1965 bp, NM_010251) (www.microrna.org). PAS = Polyadenylation site.

Several bioinformatic databases and prediction algorithms showed that the selected miRNAs (miR-155, miR-186, miR-24, miR-27b, and miR-375) have a large number of potential target genes including *Gabra4*. Several potential MREs are present along the 3′UTR of *Gabra4* (Fig.9). The minimum free energy of hybridization between each of these miRNAs and the 3′UTR of *Gabra4* is presented in Table S4. miRPath analysis predicted that specific biological pathways are induced by the upregulated miRNAs in EtOHc-4D and in AW8 neurons, including the MAPK signaling pathway that has been implicated in the effects of alcohol on the brain (Hansson et al. 2008), and that multiple target genes could be involved (Table S5).

## Discussion

This study provides evidence for a novel role for miR-186, miR-24 and/or miR-375 in mediating the effects of AW on downregulation of *Gabra4* expression in cultured mouse cortical neurons. These in vitro data will help to drive future studies using an in vivo model of AW to better understand the role of these miRNAs in alcohol addiction and their effects on *Gabra4* expression and inhibition in the brain.

### Alcohol exposure and GABA_A_R subunits

In our model system, the downregulation in *Gabra4* expression during AW (AW3-AW24), but not *Gabra1*, *Gabrd,* or *Gabrb2* at these time points suggests that the effect of AW is time-dependent and subunit specific. *Gabra1* expression showed a robust increase at AW0, AW5, and AW3. *Gabrg2* expression increased at AW24. This decrease in *Gabra4* expression during AW may lead to a decrease in the pool of *α*4-containing receptors and impact inhibition. Collectively, our results suggest that upon AW 1) there is a decrease in *α*4-mediated inhibition, 2) *Gabrg2* expression increased at AW24 to compensate for the decrease in *Gabra4* expression and 3) *α*4*βγ*2 or *α*1*βγ*2 receptors are probably the most predominant receptors in our model system. The relevance of these in vitro findings to in vivo was confirmed where we found that the mRNA level of *Gabra4* was significantly reduced at the peak of behavioral withdrawal (AW11 h) in the cortex of alcohol-withdrawn mice (Fig.3). Future studies will aim at investigating whether these molecular changes that we showed in our model system correlate with changes in excitability of cortical neurons during AW.

The expression and function of specific GABA_A_Rs subunits are differentially altered in response to acute and chronic alcohol exposure in different areas of the brain (Kumar et al. 2009). In vitro studies demonstrated that acute exposure to alcohol increased *α*4 mRNA expression levels in differentiated cortical neurons (Pignataro et al. 2007; Werner et al. 2011). For example, Pignataro et al. 2007 showed that 3 h exposure to 60 mmol/L EtOH induced an increase in *Gabra4* mRNA in differentiated cortical neurons. This was also observed in the mouse cerebral cortex where the same EtOH concentration induced a rapid, but transient increase in *Gabra4* mRNA level within 1–2 h of single intraperitoneal injection of EtOH and this level started to decrease at 24 h. Other in vitro studies showed that withdrawal of DIV15 hippocampal neurons from short-term exposure to 60 mmol/L EtOH resulted in internalization of *α*4*δ* GABA_A_Rs (Shen et al. 2011).

In vivo and in vitro studies demonstrated a remarkable plasticity of different subunits of GABA_A_Rs is response to chronic alcohol exposure and/or withdrawal. For example, mRNA and protein levels of *α*4 subunit of GABA_A_Rs increased and those of *α*1 subunit decreased in the cortex of dependent rats and alcohol-withdrawn rats (AW6-8) (Devaud et al.^1995^, ^1997^). Chronic alcohol exposure differentially altered cortical and hippocampal peptide levels of specific GABA_A_R subunits. For example, it increased *α*4 peptide levels in the hippocampus, but decreased *α*1 peptide levels in the cortex after 40 days of EtOH consumption in rats (Matthews et al.^1998^). Chronic alcohol exposure also increased *α*4-containing receptors in the cortex (Kumar et al. 2002). In the context of other GABA_A_R subunits, chronic alcohol exposure reduced *δ* subunit polypeptide levels in rat cerebellum and hippocampus but not in the rat cortex then its level reverted back to normal in both brain regions following 48 h of AW (Marutha Ravindran et al. 2007). Other researchers observed a statistically insignificant reduction in *δ* subunit polypeptide levels in cultured cerebellar granule neurons at 6 h of withdrawal from chronic alcohol exposure (AW6). This level reverted back to normal levels at AW12 (Follesa et al. 2005), consistent with our findings. In vitro studies using DIV5 rat hippocampal neurons showed that chronic exposure to EtOH (100 mmol/L/5 days) reduced *α*1 and *γ*2, but did not change *α*4 or *α*2 mRNA expression levels. Withdrawal of these neurons (AW3) from alcohol at DIV10 increased *α*4 mRNA and protein levels. mRNA levels of *α* subunits reverted back to normal at AW6 (*α*1) and AW9-12 (*α*4) (Sanna et al. 2003).

Other in vivo and in vitro studies investigated the effects of chronic intermittent ethanol treatment (CIE) and subsequent withdrawal on changes in expression and function of GABA_A_Rs. Chronic exposure to alcohol altered GABA_A_R-mediated inhibition in cortical synaptoneurosomes (Morrow et al.^1988^) and in rat cortex (Sanna et al.^1993^) and in hippocampal neurons of CIE rats (Kang et al.^1996^). Two days of withdrawal from CIE treatment resulted in modest increase in *α*4 mRNAs expression in the rat hippocampus (∼36%), thalamus (15%), and layers II/III of the cortex (12–18%) (Mahmoudi et al.^1997^). Similar treatment elevated *α*4 and *γ*2 and decreased *α*1 and *δ* peptide levels in rat hippocampus. These molecular changes correlated with a decrease in mIPSCs in CA1 pyramidal neurons and resulted in some behavioral changes (Cagetti et al. 2003). Other studies later suggested that withdrawal from CIE treatment altered the localization or trafficking of synaptic and extrasynaptic *α*4-containing receptors in rat hippocampus (Liang et al. 2006). This effect was also observed with acute ethanol exposure (Liang et al. 2007; Carlson et al. 2014). The effects of chronic ethanol exposure and/or withdrawal and CIE treatment on changes in mRNA and protein levels of GABA_A_R subunits produced different results across studies. This may be due to differences in experimental systems and in the amount, duration and timing or length of alcohol exposure and/or withdrawal. It may also be due to the pattern of alcohol exposure used (acute, chronic, or intermittent). It should be noted that changes in GABA_A_R subunits expression may not lead to functional changes in GABA_A_Rs activity during AW. Future studies will investigate if AW could alter cortical neurons excitability in GABA_A_R-dependent manner.

The results of a decade of such studies clearly indicate a pattern of remarkable plasticity of GABA_A_Rs subunits in response to alcohol exposure and also indicate that these changes are brain-region specific, multifactorial and therefore probably involve multiple mechanisms that are still not well understood.

### Alcohol exposure, *Gabra4,* and miRNAs

The transcriptional control of *Gabra4* expression as well as the precise timing of the local synthesis and insertion of the *α*4 subunit into a functional receptor at synaptic or extrasynaptic membranes can be potentially regulated by miRNAs during AW. The downregulation of *Gabra4* expression upon AW temporally correlated with the upregulation of a cohort of miRNAs that have been proposed in many studies to have physiological significance related to seizure, stress, alcoholism, epigenetics, immunity, and synaptic functions (Table S6). Our data suggest that miRNAs have specific temporal expression in response to the presence or removal of alcohol. This suggests that miRNAs have different adaptive response to alcohol exposure or withdrawal. This may be due to the intrinsic properties of each miRNA such as: 1) its rate of synthesis and degradation or turnover, 2) its protection by the RNA-Induced Silencing Complex (RISC), that is its lifespan, 3) its stability, 4) its activity (Davis and Hata 2009; Kai and Pasquinelli 2010; Ruegger and GroBhans 2012). Another explanation is that alcohol exposure and/or withdrawal may have altered the expression of specific transcription factors that regulate miRNAs expression. We could also speculate that AW may have caused epigenetic changes such as changes in DNA methylation or histone marks of miRNA genes that differentially altered the expression of these miRNAs (Miranda et al. 2010). Some of the upregulated miRNAs have been shown in several studies to be epigenetically regulated (Table S6). The overlap of 10 miRNAs in EtOHc-4D and AW neurons suggests that the effect of these miRNAs is long-lasting, and did not fade after the removal of alcohol. The effect of alcohol on other miRNAs is obviously transient. Because miRNAs are master regulators of mRNA expression and stability, we asked whether these elements could be potential candidates in causing *Gabra4* downregulation during AW. To address this, we focused on the effects of miR-155, miR-186, miR-24 and/or miR-375 on changes in *Gabra4* expression during AW. The use of chemically modified RNAs such as miRNA mimics or inhibitors to modulate miRNA expression has been proven to be a useful tool for studying the effects of these regulatory elements in a biological system (Di Martino et al. 2012; Selvamani et al. 2012). In our study, we found that mimics to miR-186 or miR-24 significantly downregulated *Gabra4* expression in AW8 neurons, which may suggest that alcohol downregulates *Gabra4* expression via these two miRNAs.

Bioinformatic analysis using several target prediction programs (Sethupathy et al. 2006) demonstrated that the upregulated miRNAs in this study have predicted MREs along the 3′UTR of *Gabra4,* but not along the 3′UTR of other GABA_A_R genes. For example, miR-186 has a conserved seed match among mammals at position 73–79 (8mer) of the 3′UTR of *Gabra4* and 3 poorly conserved seed matches at positions 180–186 (7mer-1A), 668–674 (7mer-m8), and 865–871(7mer-1A) of *Gabra4* 3′UTR. miR-24 has a poorly conserved seed match among vertebrates at position 628 (8mer), a position that is very close to the presumed binding site of miR-186 along *Gabra4* 3′UTR. miR-375 has a poorly conserved seed match among vertebrates at positions 511–517 (7mer-1A) of *Gabra4* 3′UTR. miR-155 has a potential binding site that is close to that of miR-186 and more distally located from the other miRNAs. miR-27b expression was not altered in this study, but it has a poorly conserved seed match among vertebrates at positions 331–337 (Fig.9). Poorly conserved sites can sometimes be as important as highly conserved ones in regulating gene expression (Grimson et al. 2007; Baek et al. 2008) and may point to interesting interactions of these miRNAs with the 3′UTR of *Gabra4*.

**Figure 10:**
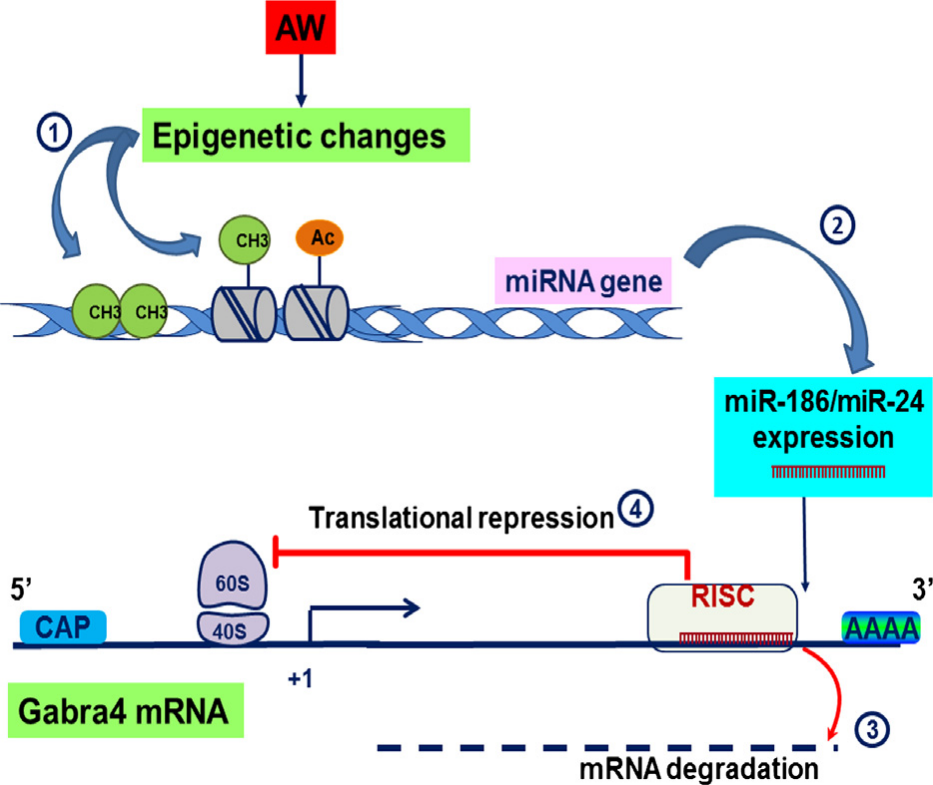
Conceptual diagram of the effects of miRNAs on Gabra4 expression during AW. The *Mus musculus* Gabra4 gene (ENSMUSG00000029211) is located on chromosome 5. The transcript (ENSMUST00000031121) is 4123 bps, has nine coding exons, 5′UTR (583 bps) and 3′UTR (1965 bps). It is translated into an alpha4 subunit (translation length = 552 residues (www.ensemble.org). 1. Alcohol withdrawal (AW) may have caused epigenetic changes in miRNA genes such as changes in DNA methylation or histone marks. 2. These alcohol-induced epigenetic changes may have altered miRNAs expression such as miR-186/miR-24. 3. Altered miRNAs could influence Gabra4 expression by binding to its 3′UTR causing mRNA degradation or 4. Translational repression or both. CH3 = methyl group; Ac = acetylation.

We experimentally confirmed that several miRNAs could regulate expression of a luciferase reporter construct that carries the 3′UTR of Gabra4. This suggests that the cumulative effects of several miRNAs are more effective on overall regulation of *Gabra4* expression than the effect of a single miRNA. Luciferase assay suggested that miR-155, miR-186, miR-24, miR-27b, and miR-375 can all bind to the 3′UTR of Gabra4. The decrease in luciferase activity correlated well with the decrease in *α*4 subunit protein levels. This may suggest that the upregulation in expression of miR-186, miR-24 and/or miR-375 during AW modulates *Gabra4* expression at the posttranscriptional level. Here, we suggest a potential molecular mechanism by which AW alters miRNAs expression and how these miRNAs may play a role in mediating the effects of AW on *Gabra4* expression (Fig.10). The complexity of the regulatory system mediated by miRNAs may be better understood in the context of other regulatory mechanisms, such as DNA methylation and histone marks that may also affect *Gabra4* expression. It will be interesting in future studies to explore the interactions among these epigenetic mechanisms in modulating *Gabra4* expression during AW.

## Conclusion

This study shows that AW downregulates *Gabra4* expression and upregulates the expression of specific miRNAs during AW. Future studies will be directed toward a possible role for these miRNAs in an in vivo model of AW. A better understanding of the regulation of *Gabra4* inhibitory function may be useful in designing strategies to use these miRNAs for the treatment of AW-related pathologies and symptoms.

## Supporting Information

**Figure S1.** Recovery of total RNAs and miRNAs from DIV15–16 control and treated neurons.

**Figure S2.** Optimization of the concentrations of mimics/inhibitors.

**Figure S3.** Subcloning of the 3′UTR of Gabra4 into a pMIR-REPORT vector.

**Table S1.** qPCR primers. **Table S2.** Changes in miRNAs following chronic exposure to 60 mM EtOH for 4 days. **Table S3.** Changes in miRNAs following withdrawal for 8 hr from chronic exposure to 60 mM EtOH for 4 days. **Table S4**. Minimum free energy of hybridization of selected miRNAs. **Table S5.** Predicted biological pathways induced by upregulated miRNAs in EtOHc-4D and AW8 neurons. **Table S6.** Functional significance of the upregulated miRNAs. **Data S1.** Dual Luciferase Assay, ELISA, and miRNA Target Prediction.
