# Supplementary figures and images for: Downregulation of Gabra4 expression during alcohol withdrawal is mediated by specific microRNAs in cultured mouse cortical neurons
Source: Brain Behav. 2015 Jun 2;5(8):e00355. doi: 10.1002/brb3.355 (PMC4559018; doi:10.1002/brb3.355)

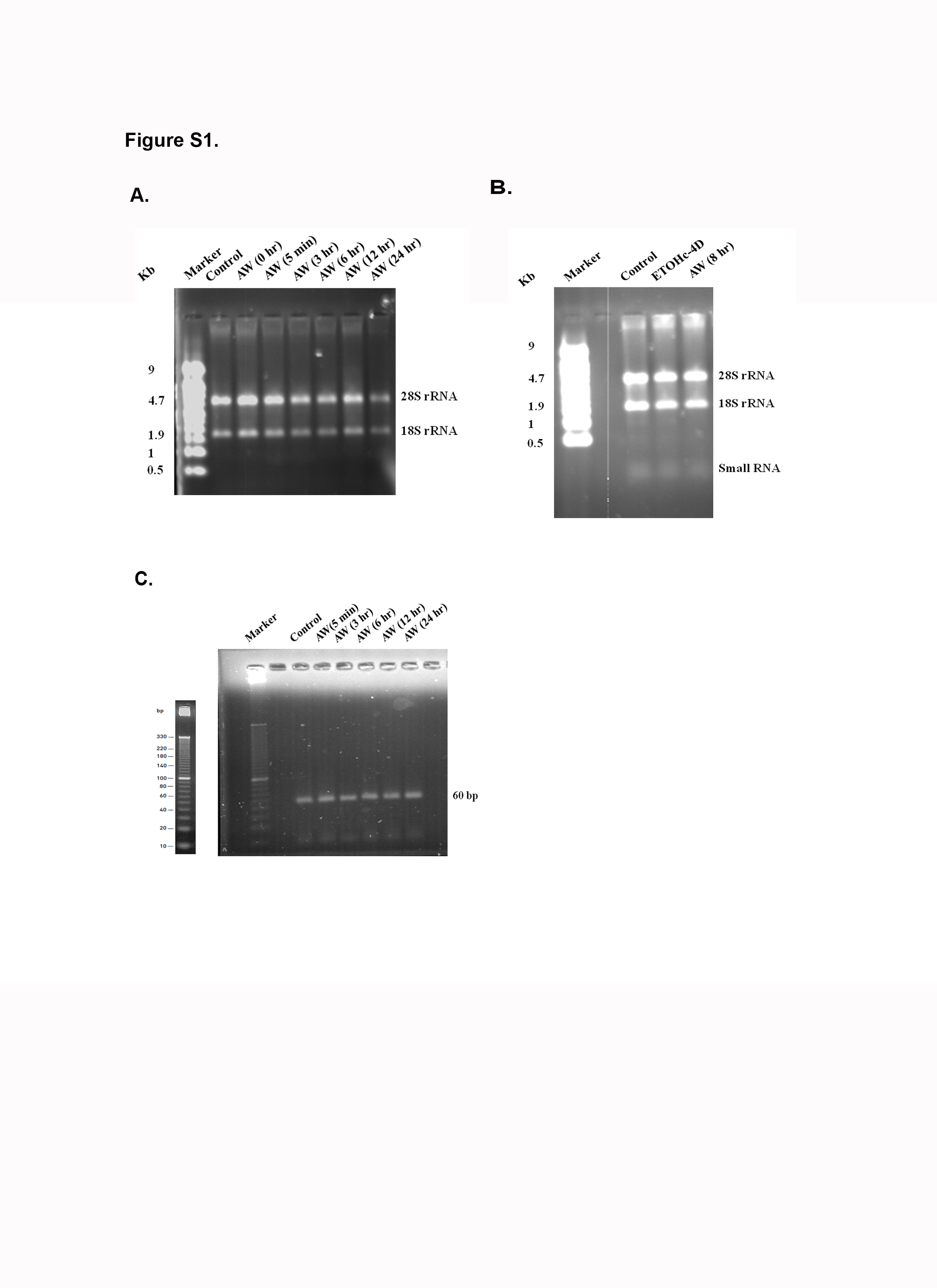

Supplement: Supplementary file 1 [file brb30005-e00355-sd1.tif]

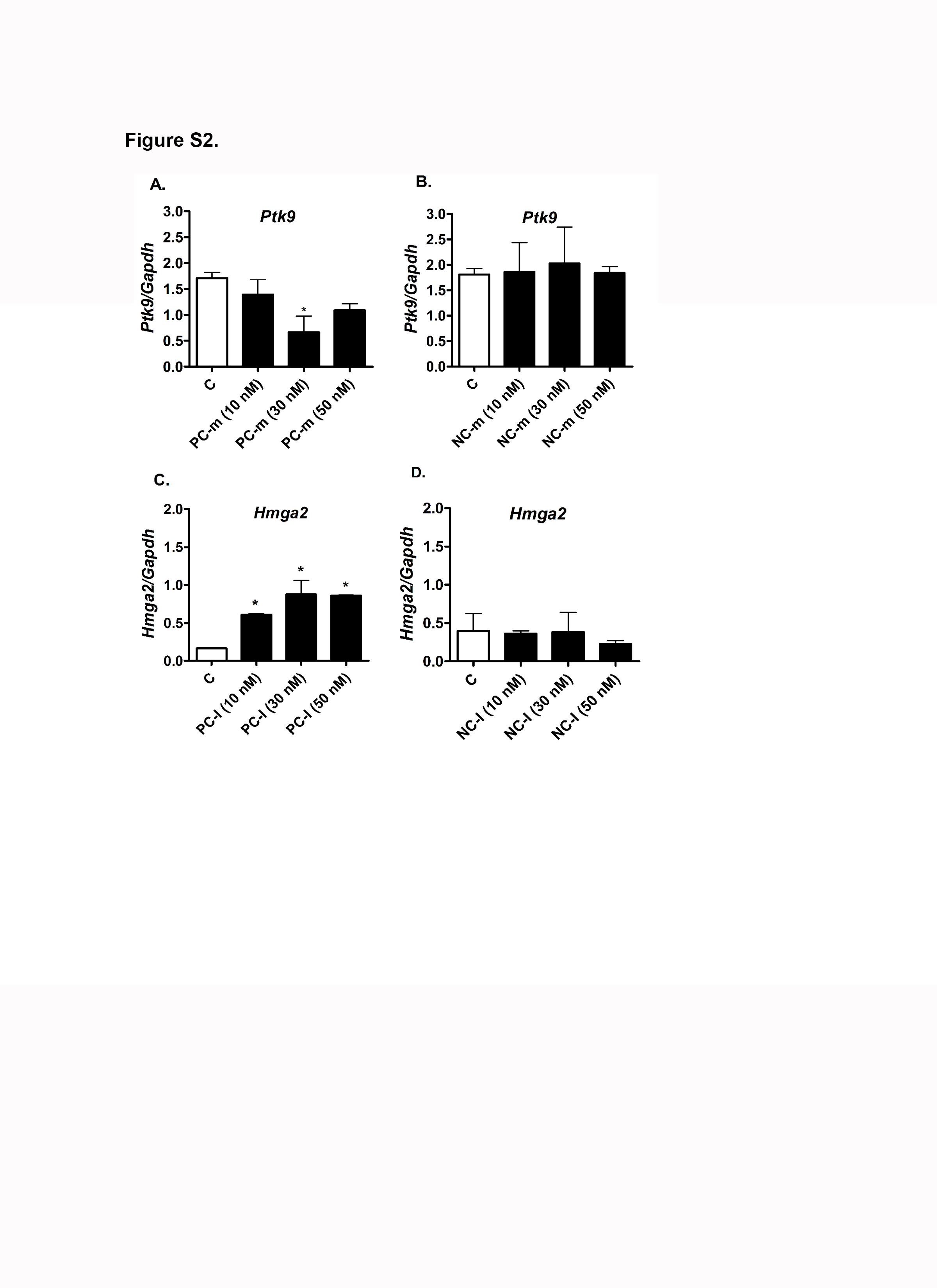

Supplement: Supplementary file 2 [file brb30005-e00355-sd2.tif]

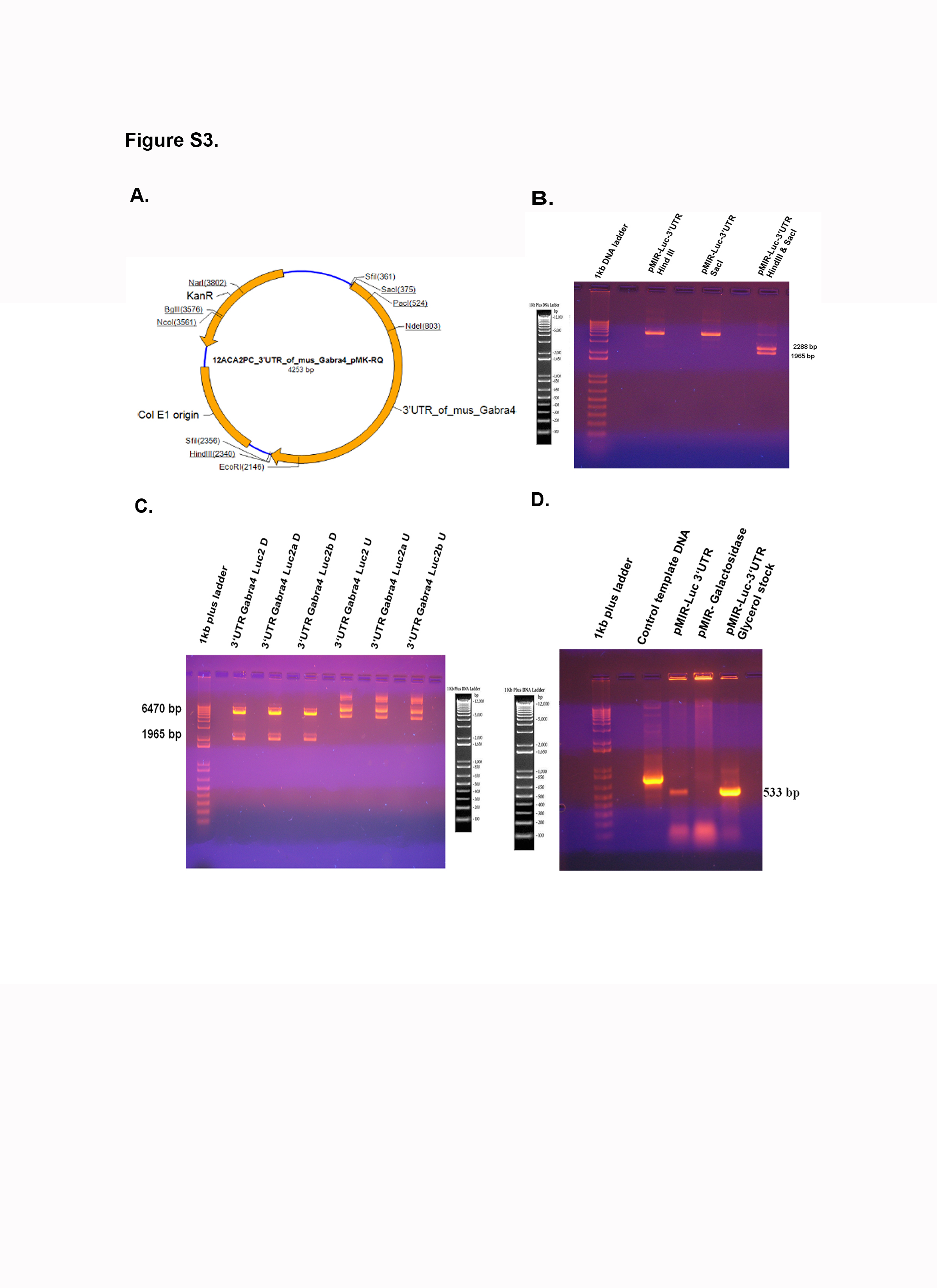

Supplement: Supplementary file 3 [file brb30005-e00355-sd3.tif]
