# Supplementary material for: Downregulation of Gabra4 expression during alcohol withdrawal is mediated by specific microRNAs in cultured mouse cortical neurons
Source: Brain Behav. 2015 Jun 2;5(8):e00355. doi: 10.1002/brb3.355 (PMC4559018; doi:10.1002/brb3.355)
Supplement: Supplementary file 4 [file brb30005-e00355-sd4.docx]

**Supplementary Tables**

**Table S1. qPCR primers**

| **Gene** | **Sequence** |
| --- | --- |
| ***Gabra4*** | F: 5'-TGAGAAGTCAGTGGAGGTGCCAAA-3'  R: 5'-TCTCGCTTGATACAGTCTGCCCAA-3' |
| ***Gabra1*** | F: 5'-ACCATGCCTAATAAGCTCCTGCGT-3'  R: 5'-CAAGTGCATTGGGCATTCAGCTCT-3' |
| ***Gabrd*** | F: 5'-TGTGGAAGTGGAGGCAAAGAAGGA-3'  R: 5'-TGCATCGATGGGTTTGAGTCTGGA-3' |
| ***Gabrg2*** | F: 5'-GTGCACTGTCCTTCAAACCTGCAA-3'  R: 5'-TAAACCCTCCCAGCTTCCACTGTT-3' |
| ***Gabrb2*** | F: 5'- ATAGCATGGAGCTCGGTCCAAAGT-3'  R:5'- ACCAGATGCTGTGTAGCAGTGACA -3' |
| ***Hmga2*** | F: 5'-AGACCGCAGTACCACTTACC-3'  R: 5'-TGGCAGTCCGAACCAAGATA-3 |
| ***Ptk9*** | F: 5'-GCCAGTTCCACCAAGCATAA-3'  R: 5'-TTGTTCGTCGTTCGCTTCC-3' |
| ***Gapdh*** | F: 5'-TCAACAGCAACTCCCACTCTTCCA-3'  R: 5'-ACCCTGTTGCTGTAGCCGTATTCA-3' |
| ***18S rRNA*** | F: 5'-TCAACTTTCGATGGTAGTCGCCGT-3'  R: 5'-TCCTTGGATGTGGTAGCCGTTTCT-3' |
| ***β-actin*** | F: 5'-TCTTGGGTATGGAATCCTGTGGCA-3'  R: 5'-ACAGCACTGTGTTGGCATAGAGGT-3' |

|  |
| --- |

**F = Forward; R= Reverse**

**Table S2. Changes in miRNAs following chronic exposure to 60mM EtOH for 4 days**

**Chronic Group – 4 Day Data (EtOHc-4D)**

| **miRNA** | **Log10 (RQ)*** | **Fold increase  from control** | **P-value** |
| --- | --- | --- | --- |
|  |  |  |  |
| **let-7b** | 0.96 | 8.9 | 0.022 |
| **let-7f** | 1.53 | 33.8 | 0.039 |
| **miR-126-3p** | 1.10 | 12.5 | 0.042 |
| **miR-126-5p** | 1.30 | 20 | 0.024 |
| **miR-152** | 1.32 | 20.6 | 0.0002 |
| **miR-155** | 1.40 | 25.3 | 0.020 |
| **miR-15b** | 1.55 | 35.5 | 0.040 |
| **miR-186** | 1.50 | 31.1 | 0.037 |
| **miR-222** | 1.12 | 13.2 | 0.048 |
| **miR-24** | 0.93 | 8.4 | 0.044 |
| **miR-31** | 0.91 | 8.2 | 0.046 |
| **miR-34c** | 0.92 | 8.2 | 0.0001 |
| **miR-375** | 0.84 | 6.9 | 0.037 |
| **miR-380-5p** | 1.05 | 11.3 | 0.028 |
| **miR-574-3p** | 0.91 | 8.1 | 0.001 |
| **miR-671-3p** | 1.19 | 15.5 | 0.003 |
| **miR-685** | 1.15 | 14.3 | 0.050 |
| **miR-7b** | 1.52 | 32.9 | 0.022 |

**Table S3. Changes in miRNAs following withdrawal from chronic exposure to EtOH**

**Withdrawal Group – 8 Hour Data (AW8)**

| **miRNA** | **Log10 (RQ)*** | **Fold**  **increase from**  **control** | **P-value** |
| --- | --- | --- | --- |
| **Let-7e** | 0.95 | 8.9 | 0.039 |
| **miR-126-3p** | 0.98 | 9.6 | 0.024 |
| **miR-126-5p** | 1.12 | 13.1 | 0.045 |
| **miR-129-3p** | 0.91 | 8.2 | 0.030 |
| **miR-151-3p** | 1.05 | 11.2 | 0.034 |
| **miR-155** | 1.25 | 18 | 0.102 |
| **miR-16** | 1.27 | 18.8 | 0.034 |
| **miR-182** | 1.04 | 11.1 | 0.013 |
| **miR-183** | 0.89 | 7.7 | 0.013 |
| **miR-186** | 0.87 | 7.3 | 0.033 |
| **miR-222** | 0.99 | 9.7 | 0.036 |
| **miR-24** | 0.76 | 5.8 | 0.024 |
| **miR-301b** | 0.88 | 7.6 | 0.039 |
| **miR-30d** | 0.96 | 9.2 | 0.012 |
| **miR-30e** | 1.08 | 12 | 0.045 |
| **miR-337-5p** | 1.18 | 15.2 | 0.029 |
| **miR-339-3p** | 0.87 | 7.4 | 0.025 |
| **miR-361** | 1.04 | 11.1 | 0.006 |
| **miR-375** | 0.92 | 8.3 | 0.009 |
| **miR-384-5p** | 0.86 | 7.2 | 0.023 |
| **miR-433** | 0.85 | 7.0 | 0.049 |
| **miR-574-3p** | 0.88 | 7.6 | 0.033 |
| **miR-671-3p** | \| 1.13 \| \| --- \| | 13.5 | 0.018 |
| **miR-685** | \| 1.10 \| \| --- \| | 12.6 | 0.001 |
| **miR-744** | 1.13 | 13.4 | 0.029 |
| **miR-770-3p** | 0.96 | 9.1 | 0.027 |
| **miR-7b** | 1.25 | 17.6 | 0.019 |
| **miR-9** | \| 0.97 \| \| --- \| | 9.3 | 0.05 |

**^*^ miRNAs with a Log10 (RQ) ≥ 0.8**

**Fold change represents the log_10_ of the average signal intensity over four arrays per group**

**Table S4.** **Minimum free energy of hybridization (mfe) of selected miRNAs**

| **TARGET : Gabra4 3’UTR (1965 bp)**  **MIRNA : mmu-miR-155-5p**  length: 23  mfe: -17.5 kcal/mol  position 236 target 5' A U 3'  CACAG AGCAUUA  GUGUU UCGUAAU  miRNA 3' UGGGGAUA AA U 5'  **7mer-m8** | **TARGET : Gabra4 3’UTR (1965 bp)**  **MIRNA : mmu-miR-186-5p**  length: 22  mfe: -15.4 kcal/mol  position 643  target 5' G UUA A U 3'  AGCC AAAG AAUUC UUG  UCGG UUUC UUAAG AAC  miRNA 3' GU CUC A 5'  **7mer-m8** |
| --- | --- |
| **MIRNA : mmu-miR-24-3p**  length: 22  mfe: -26.0 kcal/mol  position 631  target 5' A G A 3'  UG UU UGCU CUGAGCCA  AC AG ACGA GACUCGGU  miRNA 3' G A G CUU 5'  **8mer** | **MIRNA : mmu-miR-27b-3p**  length: 21  mfe: -17.1 kcal/mol  position 325  target 5' A U 3'  AGAA AG ACUGUGAA  UCUU UC UGACACUU  miRNA 3' CG GAA GG 5'  **8mer** |
| **MIRNA : mmu-miR-375-3p**  length: 22  mfe: -18.2 kcal/mol  position 490  target 5' A UA GA AG AAAG A 3'  CAC G AG GAA GAACAAA  GUG C UC CUU CUUGUUU  miRNA 3' A CG GG G 5'  **7mer-1A** | **References:**  RNAHybrid : <http://bibiserv.techfak.uni-bielefeld.de/rnahybrid/>  miRBase: <http://www.mirbase.org/mirbase.org> |

**Table S5. Predicted biological pathways induced by up-regulated miRNAs in EtOHc- 4D and AW8 neurons**

| **KEGG^a^ pathway** | **KEGG ID** | **Number of genes** | **- ln(p-value)** |
| --- | --- | --- | --- |
| **EtOHc- 4D neurons** |  |  |  |
|  |  |  |  |
| **MAPK signaling pathway** | mmu04010 | 89 | 24.85 |
| **Wnt signaling pathway** | mmu04320 | 58 | 24 |
| **Focal adhesion** | mmu04510 | 71 | 23.46 |
| **Axon guidance** | mmu04360 | 51 | 20.64 |
| **Regulation of actin cytoskeleton** | mmu04810 | 72 | 19.2 |
| **Erb signaling pathway** | mmu04012 | 37 | 18 |
| **mTOR signaling pathway** | mmu04150 | 23 | 11.75 |
| **Oxidative phosphorylation** | mmu00190 | 4 | 10.77 |
| **AW8 neurons** |  |  |  |
|  |  |  |  |
| **Axon guidance** | mmu04360 | 73 | 34.92 |
| **Regulation of actin cytoskeleton** | mmu04810 | 102 | 31.66 |
| **MAPK signaling pathway** | mmu04010 | 118 | 31.14 |
| **Focal adhesion** | mmu04510 | 91 | 25.86 |
| **Erb signaling pathway** | mmu04012 | 48 | 21.59 |
| **Wnt signaling pathway** | mmu04320 | 67 | 17.89 |
| **Oxidative phosphorylation** | mmu00190 | 6 | 15.32 |
| **Adherens junction** | mmu04540 | 43 | 12.11 |
| **T-cell receptor signaling pathway** | mmu04660 | 43 | 10.91 |

**^a^ Kyoto Encyclopedia of Genes and Genomes**

|  |  |  |  |
| --- | --- | --- | --- |
|  |  |  |  |
|  |  |  |  |
|  |  |  |  |
|  |  |  |  |
|  |  |  |  |
|  |  |  |  |
|  |  |  |  |

**Table S6. Functional significance of up-regulated miRNAs**

| **Functional categories** | **miRNAs** | **References** |
| --- | --- | --- |
|  |  |  |
| **Seizure, stroke, ischemia** | Let-7e, miR-15b, miR-126, miR-151,  **miR-155**, miR-16, miR-182, miR-183,  **miR-186, miR-24**, miR-222, miR-361,  **miR-375**, miR-30e, miR-685, miR-7b,  miR-770-3p, miR-873, let-7 | Selvamani et al. (2012) Pichardos-Casas et al. (2012)  Hunsberger et al. (2012)  Rink & Khanna (2010)  Liu et al. (2010)  Tan et al. (2009) |
| **Alcohol** | miR-10b, miR-15b, miR-152, **miR-155**,  miR-183, **miR-186, miR-24**, miR-339,  **miR-375**, miR-380, miR-34c, miR-7b,  miR-685, let-7f, miR-9 | Lippai et al. (2013)  Tapocik et al. (2012)  Nunez & Mayfield (2012)  Guo et al. (2012)  Lewohl et al. (2011) Avissar et al. (2009) |
| **Synapses** | Let-7, miR-153, **miR-155**, miR-182,  miR-183, **miR-186**, miR-380, miR-34, miR-7, miR-9 | Wang et al. (2013)  Yu et al. (2012)  Doxakis (2010)  Lugli et al. (2008) |
| **Epigenetics** | miR-129, miR-152, **miR-155**,miR-183,  **miR-24**, miR-30d, miR-34c, **miR-375**,  miR-7b | Guo et al. (2012)  Sato et al. (2011)  Tsuruta et al. (2011) Wu et al. (2010) |
| **Stress** | miR-10b, let-7, miR-7b, miR-183, **miR-186**,  miR-9, **miR-24** | Nadorp & Soreq (2014)  Zucchi et al. (2013)  Babenko et al. (2012)  Rinaldi et al. (2010)  Meerson et al. (2010)  Lee et al. (2006) |
| **Immunity** | Let-7, miR-152, **miR-155,** miR-15b, miR-16, miR-31, miR-9 | Lopez-Ramirez et al. (2014)  Nunez & Mayfield (2012)  Yamagishi et al. (2012)  Cardoso et al. (2012)  Liu et al. (2010)  Bazzoni et al. (2009) |
| **Cell cycle**  **Neurite outgrowth** | miR-16, miR-129, **miR-24**, miR-222 | Wu et al. (2010)  Bandi et al. (2009)  Le Sage et al. (2007) |
|  | **miR-375**, miR-7 | Abdelmohsen et al. (2010)  Chen et al. (2010) |
|  |  |  |

**Data S1**

***Dual Luciferase Assay***

pMIR-Gal, pMIR-Luc and pMIR-Luc-3’UTR plasmids were extracted from transformant cells using the Purelink Hipure plasmid DNA purification kit (Life Technologies, Invitrogen) following the manufacturer’s instructions. Four independent neuronal cultures were used for the luciferase assay. Transfection of cortical neurons with plasmid and/or miRNA mimics to
miR-155, miR-186, miR-24, miR-27b or miR-375 was performed at DIV8. 2.5 µg of plasmid of pMIR-Gal + pMIR-Luc, 2.5 µg of pMIR-Gal + pMIR-Luc-3’UTR with or without miRNA mimic (30 nM) or with scrambled oligo (30 nM) were diluted in 500 µl Opti-MEM I reduced serum media (Life Technologies, Invitrogen) and incubated at RT for 5 min. 100 µl of the mixture was used per well. Transfection of cortical neurons was performed using 8 µl of Lipofectamine 2000 reagent (Life Technologies, Invitrogen) in 100 µl Opti-MEM I reduced serum media (Life Technologies, Invitrogen) for 5 min at RT per well following the manufacturer’s instructions. This mixture was then combined with the 100 µl of diluted plasmid at RT for 20 min before transfection. Cultured cortical neurons in a 12-well plate were co-transfected at DIV8 for 2-3 hr with 100 µl/well of the mixture containing the plasmid and the Lipofectamine 2000. Non-transfected (NT) neurons were used to assess the efficiency of plasmid transfection. After 3 hr transfection, the transfection medium was removed and replaced with conditioned medium. Neurons were then maintained for 24 hr at 37^0^C. Luciferase activity was analyzed 24 hr post-transfection (Ma et al. 2004). Luciferase assay was performed using the Dual-Light System (Life Technologies, ABi) following the manufacturer’s instructions and the Infinite 200 microplate reader (Tecan). Triplicate samples from NT and transfected neurons were used and the average values of luciferase or galactosidase light units were obtained. The luciferase activity was determined as the ratio of the average of luciferase light units generated by each sample divided by the average of the galactosidase light units (Life Technologies, ABi).

***Enzyme-linked immunoabsorbant assay***Briefly, we used the lysates that we collected for the luciferase assay. We constructed a standard curve using a standard that is supplied with the kit. Lysates from all samples were added in duplicates to a 96-well plate that is coated with a highly sensitive and specific antibody that recognizes the mouse Gabra4 protein. The capture Ab is a mouse monoclonal and the detection Ab is a goat polyclonal Ab. Non-specific binding wells were included in this assay. After 2 hr incubation at 37^0^C, a biotin antibody then an HRP-avidin was added to each well. After several washes, a TMB substrate was added to each well then followed by a Stop solution. Samples values were read at 450 nm using the FlexStation 3 Multi-Mode microplate reader (Molecular Devices) and the SoftMax Pro v5.2.

***miRNAs Target Prediction***miRNAs that show significant difference in expression (P<0.05) by miRNA microarray analysis and by qRT-PCR in EtOHc-4D and AW8 neurons were selected for target prediction. Since the down-regulation of *Gabra4* correlated with an up-regulation of specific miRNAs, we used several bioinformatic databases (Hammell 2010) to search whether any of these up-regulated miRNAs have predicted binding sites along the 3’UTR of the *Mus musculus* *Gabra4.* The well-established algorithms such as TargetscanMouse ([www.targetscan.org](http://www.targetscan.org)) (Grimson et al. 2007), and miRanda ([www.microrna.org](http://www.microrna.org)) (Betel et al. 2008), were used since they provide information about the conservation among species of the miRNA binding site (MRE) along the 3’UTR of the target gene. We also used RNAhybrid (<http://bibiserv.techfak.uni-bielefeld.de/rnahybrid/>) (Rehmsmeier et al. 2004) which uses a thermodynamic approach to find the most energy favorable hybridization sites between miRNA and the 3’UTR of the target gene. miRDB ([www.mirdb.org](http://www.mirdb.org)) was also used (Wang et al. 2008). The integration of various computational methods indicated that miR-155, miR-186, miR-24, miR-27b and miR-375 have predicted binding sites on the 3’UTR of the *Mus musculus* Gabra4 (1965bp, NM_010251) (Fig. 9). For example, miR-186 targets the 3’UTR of the *Mus musculus* *Gabra4* gene at 4 different positions. miR-186 has a conserved seed match at positions 73-79 (8 mer) and three poorly conserved seed matches at positions 180-186 (7mer-1A), 668-674 (7mer-m8), and 865-871(7mer-1A) of the 3’UTR of the *Mus musculus* *Gabra4*. Moreover, miR-24 at positions 640-646 (8mer), miR-155 at two positions 241-247 (7mer-m8) and 1055-1059 (7mer-m8), miR-27b at position 331-337 (8mer) and miR-375 at positions 512-518 (7mer-1A) have poorly conserved seed matches with the 3’UTR of *Gabra4*. Table S4 shows the minimum free energy of hybridization (mfe) in Kcal/mole between the selected miRNAs (miR-155, miR-186, miR-24, miR-27b and miR-375) and the 3’UTR of Gabra4.

We also determined the predicted biological pathways that are regulated by the differentially expressed miRNAs in EtOHc-4D and AW8 neurons using the DIANA-miRNApath (<http://diana.cslab.ece.ntua.gr/pathways/>) search tool. The pathways that have the largest number of genes that are affected by miRNAs with the highest –ln (p-value) are listed in Table S5.

**Supplementary Legends**

**Figure S1. Recovery of total RNAs and smaller RNAs from DIV15-16 control and treated neurons. (A)** A 1.5% agarose gel electrophoresis shows the recovery of the 18S and 28S rRNA bands from control (lane 2) and AW neurons (lanes 3-8). **(B)** A 1.5% agarose gel shows the recovery of the 18S and 28S rRNA as well as smaller RNAs from control (lane 3), ETOHc-4D (lane 4) and AW (8 hr) neurons (lane 5) for the array experiment. Lane 1=RNA Millennium marker in Kb. **(C)** A representative picture of 5% agarose gel that shows the amplified miR-155 (60 bp), using the stem loop real-time polymerase chain reaction, in control (Lane 3) and AW neurons (Lanes 4-8). Lane 1=10 bp ladder.

**Figure S2. Optimization of the concentrations of mimics/inhibitors.** **(A&B)** Changes in *Ptk9* expression in response to 10, 30, or 50 nM of miR-1 positive control mimic (PC-m) or negative control mimic (NC-m). (*P<0.05 PC-m (30 nM) compared to control C). **(C&D)** Changes in *Hmga2* expression in response to 10, 30 or 50 nM of Let-7c positive control inhibitor (PC-I) or negative control inhibitor (NC-I). (*P<0.05 PC-I 10, 30 or 50 nM compared to control C).

**Figure S3. Cloning of the 3’UTR of the *Mus musculus* Gabra4 into a pMIR-REPORT vector. (A)** Cloning of the 3’UTR of Gabra4 into a pMK-RQ vector (4253 bp). The 3’UTR is flanked by SacI and HindIII at the 5’ and 3’- ends. **(B)** Digestion of pMK-RQ with HindIII, SacI or both then the digests were run on 1% agarose gel. Lane 1 shows the 1Kb ladder, lanes 2 & 3 show a single band around 4 kb and lane 4 shows 2 bands (2288bp and 1965 bp). **(C)** Subcloning of the 3’UTR of Gabra4 into a pMIR-REPORT vector. This vector was digested by HindIII/SacI then the digests were run on 1% agarose gel. Lane 1 shows the 1Kb ladder, lanes 2, 3 & 4 show two bands (6470bp & 1965 bp). Lanes 4, 5 & 6 show the undigested vector **(D)** Successful transformation of TOP10 *E.coli* competent cells with the α4 3’UTR vector was

assessed by PCR. Lane 1=1Kb ladder, lane 2=control template DNA, Lanes 3 & 5 show a band around 533 bp which got amplified by the Gabra4 3’UTR primer, Lane 4= negative control showing no amplification of a product from Gal colonies using the Gabra4 3’UTR primer.

**Table S1.** **qPCR primers**. The sequences of the forward and reverse qPCR primers.

**Table S2. Changes in miRNAs gene expression following chronic exposure to 60 mM EtOH for 4 days.** RQ represents the fold change of miRNAs in neurons that were chronically exposed at DIV11 to 60 mM EtOH for 4 days (EtOHc-4D). N=4 control neurons and N=4 chronic neurons. P<0.05 is considered statistically significant using the Student’s t-test.
**Table S3. Changes in miRNAs gene expression following withdrawal for 8 hr from chronic exposure to 60 mM EtOH for 4 days.** RQ represents the fold change of miRNAs in neurons that were withdrawn at DIV15 for 8 hr (AW8) from chronic exposure to 60 mM EtOH for 4 days. N=4 control neurons and N=4 AW neurons. P<0.05 is considered statistically significant using the Student’s t-test.

**Table S4**. **Minimum free energy of hybridization (mfe) of selected miRNAs**. Minimum free energy of hybridization (mfe) in Kcal/mole between miRNAs and the 3’UTR of Gabra4, calculated using RNAHybrid.

**Table S5**. **Predicted biological pathways induced by up-regulated miRNAs in EtOHc-4D and AW8 neurons.** DNA Intelligent Analysis (DIANA) results showed that eight predicted biological pathways in EtOHc-4D neurons and nine in AW8 neurons are induced by the up-regulated miRNAs. KEGG ID, number of genes found and the –ln(p-value) are shown. We only selected the pathways showing a –ln(p-value) ≥ 10.

**Table S6. Functional significance of up-regulated miRNAs.** miRNA microarray showed the up-regulation of a subset of physiologically relevant miRNAs.

**Supplementary References**

Abdelmohsen, K., E.R. Hutchison, E.K. Lee, Y. Kuwano, M.M. Kim, K. Masuda, et al. 2010. miR375 inhibits differentiation of neuritis by lowering HuD levels. Mol. Cell Biol. 30(17): 4197-4210.

Avissar, M., M.D. McClean, K.Y. Kelsey, C.J. Marsit. 2009. MicroRNA expression in head and neck cancer associates with alcohol consumption and survival. Carcinogenesis. 30(12): 2059-2063.

Babenko, O., A. Golubov, Y. Ilnytskyy, I. Kovalchuk, G. Metz. 2012. Genomic and epigenomic responses to chronic stress involve miRNA-mediated programming. PLoS One. 7(1): 1-17.

Bandi, N., S. Zbinden, M. Gugger, M. Arnold, V. Kocher, L. Hasan, et al. 2009. miR15a and miR16 are implicated in cell cycle regulation in Rb-dependent manner and are frequently deleted or down-regulated in non-small cell lung cancer. Cancer. 69 (13): 5553- 5559.

Bazzoni, F., M. Rossato, M. Fabbri, D.Gaudiosi, M. Mirolo, L. Mori, et al. 2009. Induction and regulatory function of miR9 in human monocytes and neutrophils exposed to proinflammatory signals. PNAS. 106: 5282-5287.

Betel, D., M. Wilson, A. Gabow, D.S. Marks, C. Sander. 2008. The microrna.org resource: targets and expression. Nucleic Acids Res. 36: D149-153.

Cardoso, A.L., J.R. Guedes, L. Pereira de Almeida, M.C. Pedroso de Lima. 2012.
miR-155 modulates microglia-mediated immune response by down-regulating SOCS-1 and promoting cytokine and nitric oxide production. Immunology. 135(1): 73-88.

Chen, H., R. Shalom-Feuerstein, J. Riley, S.D. Zhang, P. Tucci, M. Agostini, et al. 2010. miR7 and miR124 are specifically expressed during neuroblastoma differentiation, cortical development and embryonic stem cells differentiation and control neurite outgrowth in vitro. Biochem. Biophys. Res. Commun. 394: 921-927.

Doxakis, E. 2010. Post-transcriptional regulation of alpha-synuclein expression by miR7 and miR153. J. Biol. Chem. 285:12726-12734.

Grimson, A., K.K. Farh, W.K., Johnston, P. Garrett-Engele, L.P. Lim, and D.P. Bartel. 2007. MicroRNA targeting specificity in mammals determinants beyond seed pairing. Mol. Cell. 27: 91-105.

Guo, Y., Y. Chen, S. Carreon, M. Qiang. 2012. Chronic intermittent ethanol exposure and its removal induce a different miRNA expression pattern in primary cortical neuronal cultures. Alcohol. Clin. Exp.Res. 36(6):1058-1066.

Hammell, M. 2010. Computational methods to identify miRNAs targets. Semin. Cell Dev. Biol. 21(7): 738-744.

Hunsberger, J.G., E.B. Fessler, Z. Wang, A.G. Elkahloun, D.M. Chuang. 2012. Post-insult valproic acid-regulated microRNAs: potential targets for cerebral ischemia. Am. J. Transl. Res. 4(3): 316-332.

Lee, H., M. Palkovits, W.S. Young. 2006. miR7b, a microRNA up-regulated in the hypothalamus after chronic hyperosmolar stimulation, inhibits Fos translation. PNAS. 103(42):15669-15674.

Le Sage, C., R. Nagel, D.A. Egan, M. Schrier, E. Mesman, A. Mangiola, et al. 2007. Regulation of the p27(kip1) tumor suppressor by miR221 and miR222 promotes cancer cell proliferation. EMBO J. 26(15): 3699-3708.

Lippai, D., S. Bala, T. Csk, E.A. Kurt-Jones, G. Szabo. 2013. Chronic alcohol-induced microRNA-155 contributes to neuroinflammation in a TLR4-dependent manner in mice. PLoS One. 8(8): e70945.

Liu, D., Y. Tian, B.P. Ander, H. Xu, B.S. Stamova, X. Zhan, et al. 2010. Brain and blood microRNA expression profiling of ischemic stroke, intracerebral hemorrhage and kainate seizures. J. Cereb. Blood Flow Metab. 30:92-101.

Liu, X., Z. Zhan, L. Xu, F. Ma, D. Li, Z. Guo, et al. 2010. MicroRNA-148/152 impair innate response and antigen presentation of TLR-triggered dendritic cells by targeting CamKIIα. J. Immunology. 185:7244-7251.

Lopez-Ramirez, M.A., D. Wu, G. Pryce, J.E. Simpson, A. Reijerkerk, J. King-Robson, et al. 2014. MicroRNA-155 negatively affects blood-brain barrier function during neuroinflammation. FASEB J. 28 (6): 2551-2565.

Lugli, J., V.I. Torvik, J. Larson, N.R. Smalheiser. 2008. Expression of microRNAs and their precursors in synaptic fractions of adult mouse forebrain. J. Neurochem. 106: 650-661.

Meerson, A., L. Cacheaux, K.A. Goosens, R.M. Sapolsky, H. Soreq, D. Kaufer. 2010. Changes in brain microRNAs contribute to cholinergic stress reactions. J. Mol. Neurosci. 40(1-2): 47-55.

Nadorp, B., and H. Soreq. 2014. Predicted overlapping microRNA regulators of acetylcholine packaging and degradation in neuroinflammation-related disorders. Front. Mol. Neurosci. 7:1-11.

Nunez, Y. and R. Mayfield. 2012. Understanding alcoholism through microRNA signatures in brains of human alcoholics. Front. Genetics. 3:1-13.

Pichardo-Casas, I., L. Goff, M. Swerdel, A. Athie, J. Davila, M. Ramos-Brossier, et al. 2012. Expression profiling of synaptic microRNAs from the adult rat brain identifies regional differences and seizure-induced dynamic modulation. Brain Res. 1436:20-33.

Rehmsmeier, M., P. Steffen, M. Hochsmann, R. Giegerich. 2004. Fast and effective prediction of miRNA/target duplex. RNA. 10:1507-1517.

Rinaldi, A., S. Vincenti, F. De Vito, I. Bozzoni, A. Oliverio, C. Presutti, et al. 2010. Stress induces region specific alterations in microRNAs expression in mice. Behavior & Brain Res. 208:265-269.

Rink, C., S. Khanna. 2010. MicroRNA in ischemic stroke etiology and pathology. Physiol. Genomics. 43(10):512-528.

Sato, F., S. Tsuchiya, S. Meltzer, K. Shimizu. 2011. MicroRNAs and epigenetics. FEBS J. 278:1598-1609.

Selvamani, A., P. Sathyan, R. Miranda, F. Sohrabji. 2012. An antagomir to microRNA let7f promotes neuroprotection in an ischemic stroke model. PLoS One. 7(2): e32662; 1-11.

Tan., K.S., A. Armugam, S. Sepramaniam, K.Y. Lim, K.D. Setyowati, C.W. Wang, et al. 2009. Expression profile of microRNAs in young stroke patients. PLoS One. 4(11): e7689.

Tsuruta, T., K.I. Kozaki, A. Uesegi, M. Furuta, A. Hirasawa, I. Imoto, et al. 2011. miR152 is a tunor suppressor microRNA that is silenced by DNA hypermethylation in endometrial cancer. Cancer Res. 71:6450-6462.

Wang, X., Y. Zhao, X. Zhang, H. Badie, Y. Zhou, Y. Mu, et al. 2013. Loss of soring nexin 27 contributes to excitatory synaptic dysfunction by modulating glutamate receptor recycling in Down’s syndrome. Nat. Med. 19(4): 473-480.

Wang, X. 2008. miRDB: a microRNA target prediction and functional annotation database with a wiki interface. RNA. 14:1012-1017.

Wu, J., J. Qian, C. Li,L. Kwok, F. Cheng, P. Liu, et al. 2010. miR129 regulates cell proliferation by downregulating Cdk6 expression. Cell cycle. 9(9):1809-1818.

Yamagishi, M., K. Nakano, A. Miyake, T. Yamochi, Y. Kagami, A. Tsutsumi, et al. 2012. Polycomb-mediated loss of miR31 activates NIK-dependent NF-KB pathway in adult T cell leukemia and other cancers. Cancer Cell. 21(1):121-135.

Yu, B., T. Qian, Y. Wang, S. Zhou, G. Ding, F. Ding, et al. 2012. miR182 inhibits Schwann cell proliferation and migration by targeting FGF9 and NTM, respectively at an early stage following sciatic nerve injury. Nucleic Acids Res. 40(20):10356-10365.

Zucchi, F.C.R., Y. Yao, I.D. Ward, Y. Ilnytskyy, D.M. Olson, K. Benzies, et al. 2013. Maternal stress induces epigenetic signatures of psychiatric and neurological diseases in the offspring. PLoS One. 8(2): e56967.
